# ﻿Ancient reproductive modes and criteria of multicellularity

**DOI:** 10.3897/compcytogen.17.109671

**Published:** 2023-10-20

**Authors:** Ilya A. Gavrilov-Zimin

**Affiliations:** 1 Zoological Institute, Russian Academy of Sciences, Universitetskaya nab. 1, St. Petersburg, 199034, Russia Zoological Institute, Russian Academy of Sciences St. Petersburg Russia

**Keywords:** Evolution, gametogenesis, multicellularity, oogamete, polyembryony, sexual and asexual reproduction, spore, viviparity

## Abstract

It is demonstrated that the initial method of fertilization in animals (Metazoa), embryophyte plants (Embryophyta), most groups of multicellular oogamous algae, oogamous and pseudoogamous multicellular fungi was internal fertilization (in the broad meaning) in/on the body of a maternal organism. Accordingly, during the bisexual process, the initial method of formation of a daughter multicellular organism in animals was viviparity, and in embryophyte plants and most groups of oogamous multicellular algae – the germination of a zygote in/on the body of maternal organism.

The reproductive criteria of multicellularity are proposed and discussed. In this regard, the multicellularity is considered to subdivide terminologically into three variants: 1) protonemal, the most simple, characteristic of multicellular prokaryotes, most groups of multicellular algae and gametophytes of some higher plants; 2) siphonoseptal, found among multicellular fungi, some groups of green and yellow-green algae; 3) embryogenic, most complicated, known in all animals (Metazoa), all sporophytes and some gametophytes of higher plants (Embryophyta), charophyte green algae Charophyceae s.s., oogamous species of green and brown algae, some genera of red algae.

In addition to the well-known division of reproduction methods into sexual and asexual, it is proposed to divide the reproduction of multicellular organisms into monocytic (the emergence of a new organism from one cell sexually or asexually) and polycytic (fragmentation, longitudinal / transverse division or budding based on many cells of the body of the mother organism), since these two ways have different evolutionary and ontogenetic origins.

## ﻿Introduction

The origin of multicellularity in the evolution of living organisms remains one of the most important discussion topics in evolutionary biology over the past one and a half centuries. The main hypotheses explaining the sequential phylogenetic transformation of colonial protists into the first truly multicellular organisms are well known and discussed many times in specialized scientific and educational literature (see, for example, Zakhvatkin 1949, 1956; Ivanov 1968; Ivanova-Kazas 1995; Bonner 1998; Grosberg and Stratchmann 2007; Michailov et al. 2009; Knoll 2011; Herron et al. 2013; Niklas and Newman 2013; Suga and Ruiz-Trillo 2013; Umen 2014; Coates et al. 2015; Brunet and King 2017; Malakhov et al. 2019; Colizzi et al. 2020; Lamża 2023, etc.). In these hypotheses and the discussions accompanying them, the main place is given to morpho-anatomical, ontogenetic and molecular changes, without which the transition from the simple unicellular level of life organization to a higher level is impossible. At the same time, the question of how exactly the reproduction of the first multicellular organisms could be carried out is given much less attention, and some important aspects are completely overlooked. However, a clear answer to this question is necessary to understand the entire course of the subsequent evolution of reproductive systems. In addition, as will be shown below, the features of reproduction can be considered as the important criteria for multicellularity itself.

The traditional, well-known division of reproduction modes into two large groups, sexual and asexual, has an almost universal meaning, since it is to some extent applicable to all living systems, with the exception of only prokaryotic organisms and viruses. To avoid confusion, it should be noted right away that asexual and sexual methods of reproduction are not always accompanied by the increasing of a population. For example, in higher plants (Embryophyta), as well as in most groups of algae and fungi, producing of numerous descendants occurs primarily with the asexual formation of spores, while as a result of the sexual process, only one daughter organism (usually a sporophyte) often develops on one maternal organism (usually a gametophyte), that is, there is no increase in the number of individuals. The sexual process in prokaryotic organisms and in some protists is not at all directly connected with reproduction.

Significant terminological confusion also occurs when discussing variants of parthenogenesis, i.e. development of an organism from a gamete without its fusion with another gamete. In recent decades, especially in the English-language literature (see, for example, Heesch et al. 2021), it has become commonplace to attribute parthenogenesis to asexual reproduction. With this approach, the difference between asexual and sexual reproduction is made dependent on a random event (fusion of gametes), which may not occur in the life cycle of an individual for external reasons that do not depend on its morphology, physiology, lifestyle, taxonomic and phylogenetic position. That is, the classification of a biological phenomenon (reproduction) in this case is made dependent on random non-biological causes. This approach could theoretically be justified by the homology and great similarity between the development of the unfertilized gamete and the spore in many simply constructed organisms. However, in all higher plants, oogamous algae, and all animals, gametogenesis usually differs sharply from the processes of asexual reproduction and is associated with the spatial and functional separation of the germ cell line from somatic ones. In this regard, the traditional approach to understanding parthenogenesis as a variant of sexual reproduction seems more convenient, since parthenogenetic offspring arise from an extremely specialized haploid germ cell – the gamete, which, moreover, in many cases merges with one or another other product of gametogenesis to restore its diploidy (for example, with polar bodies). In plant organisms, parthenogenesis itself should, of course, be distinguished from other variants of apomixis, in which the embryo arises not from the egg, but from other cells of the embryo sac, nucellus, or integument (see: Yakovlev 1981: 7–8; Reproductive Systems 2000: 142–218).

In addition, when considering methods of reproduction of multicellular organisms, it is important not to lose sight of the following aspect. A daughter multicellular organism can arise from a single cell of the mother’s body (spore, zygote, haploid gamete, parthenogenetic egg with restored diploidy, or simply a separate somatic cell that has retained totipotency [that is, the ability to produce various types of differentiated cells]) or simultaneously from many mother cells (with various variants of budding, fragmentation, simple division of the body into two or many parts). According to this criterion, the reproduction of multicellular organisms can be divided into monocytic and polycytic; the second term only partly overlaps with the concept of “vegetative reproduction”, since in the botanical literature, simple mitotic division of unicellular algae is also called vegetative (see, for example, Belyakova et al. 2006b) and various cases of budding based on one initial meristematic cell (Reproductive Systems 2000: 342). In different senses, vegetative reproduction is also mentioned in the zoological literature (Ivanova-Kazas 1977). The term “blastogenesis” is closer in meaning to polycytic reproduction, which is understood as the opposite of embryogenesis (Ivanova-Kazas 1977: 227) and corresponds to polycytic budding (see below). As will be shown below, the division of reproduction into monocytic and polycytic is no less important for understanding the evolution of reproduction and self-reproduction than the criterion for the presence/absence of gamete fusion.

Numerous taxonomic names of organisms are used in the analysis below. It is important for the reader who does not have a serious personal experience of taxonomic work to take into account that there is no single universal system of living nature and a universal method of taxonomic constructions. For any group of organisms, the scientific literature presents competing views of various specialists and scientific schools on the phylogeny of the corresponding group and its “internal” classification. At the same time, phylogenetic schemes and taxonomic systems published later in date are by no means necessarily more correct or more reasoned than those published earlier. In this article, I do not have the opportunity to discuss any particular aspects of phylogenesis, the ideological basis of numerous classification schemes, contradictions between evolutionary and cladistic systematics, the suitability/unsuitability of various computer-molecular approaches, etc. Solely for practical convenience, I use the names of algal taxa appearing in the AlgaeBase database (https://www.algaebase.org/), since this database compiles all nominal taxa of algae (as well as cyanobacteria) at the same time and reveals the corresponding nomenclature of names. The use of AlgaeBase does not mean my automatic agreement with all classification constructions implemented in this database. The same applies to the use of the names of higher taxa of heterotrophic protists and invertebrate animals, the classifications of which differ quite significantly in the works of different authors published in recent decades. In general, I follow the approach used in one of the most famous modern manuals on invertebrate zoology, a two-volume edition edited by Westheide and Rieger (Westheide and Rieger 2004). Unlike later papers (e.g., Dunn et al. 2014), which claim to reconstruct the phylogeny and provide a general classification of animals, this fundamental guide differs in that it is based primarily on easily verifiable and well-studied phenotypic characters of organisms. When using the names of higher taxa of terrestrial plants (Embryophyta) and fungi, I am guided by the multi-volume monograph “Botanica”, prepared by a team of specialists from the Faculty of Biology of Moscow State University (Belyakova et al. 2006a, b; Timonin 2007; Timonin and Filin 2009; Timonin et al. 2009).

## ﻿Reproductive criteria of multicellularity

For further discussions, it is necessary to clearly define the range of organisms that can be considered multicellular. Unfortunately, the border between the coloniality of unicellular protists and simple forms of multicellularity is understood in the scientific literature very vaguely. With an expanded approach to this issue (for example, Grosberg and Stratchmann 2007), multicellular organisms, in addition to animals, higher plants and a number of groups of algae, also mean some groups of slime molds and fungi, as well as a number of groups of Prokaryota.

In addition, there is no clear unequivocal separation of different types of multicellularity. Usually, one speaks only of simple and complex multicellularity (Knoll 2011; Niklas and Newman 2013), implying the presence of differentiated cells and tissues by the latter. However, the degree of differentiation varies greatly from one taxon to another (and even between individual stages of the life cycle of the same species of organisms) and demonstrates numerous chaotic transitions from simpler to more complex options and back.

In a broad interpretation, “clonal” and “aggregative” multicellularity are also distinguished (Grosberg and Stratchmann 2007; Coates et al. 2015; Lamża 2023), meaning by the latter the formation of cell clusters from the original free-living unicellular organisms. This approach seems to me unfortunate, since it does not allow any clear distinction between the various colonial prokaryotes, colonial fungi and algae, on the one hand, and the multicellular representatives of these same groups, on the other hand.

**Figure 1.**
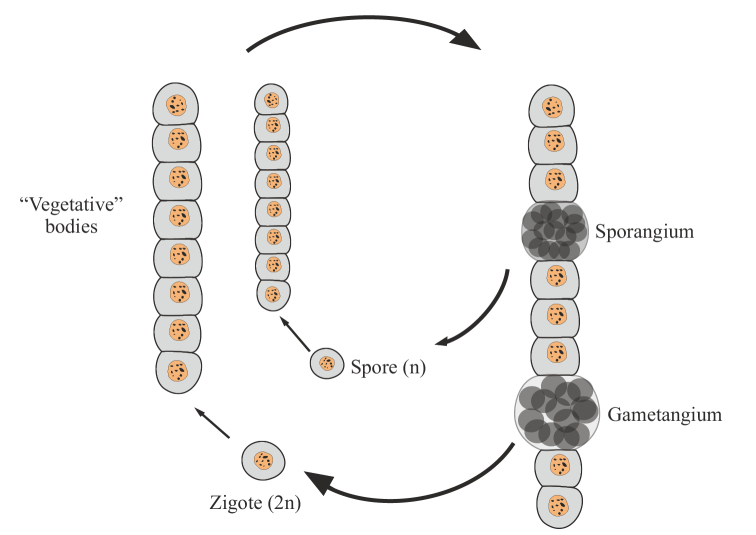
Generalized scheme of the life cycle of a multicellular organism (protonemal multicellularity).

I consider it logical to proceed from the fact that a unitary multicellular organism, unlike a colonial one, obligatorily develops as a multicellular organism and reproduces itself only after it reaches the multicellular «vegetative» stage of ontogenesis. That is, the life cycle of a unitary multicellular organism is as follows (Fig. 1). In such a cycle, the only unicellular (and mononuclear for eukaryotes) stage is the spore, gamete, or zygote that has no an independent life (i.e., nutrition, reproduction). A multicellular body of a unitary organism obligately grows from a spore, zygote or parthenogenetic gamete. This first reproductive criterion for multicellularity avoids ambiguity in the understanding of coloniality vs. unitary multicellularity and adequately assess the evolutionary consequences of the transition from one level of life organization to another. In particular, different variants of colonies in archaea (Archaea), myxobacteria (Mixococcales) and slime molds (Myxomycota, Acrasiomycota), even in the most complex cases, are only secondary accumulations of independent cells homogeneous in structure or multinucleated plasmodia, pseudoplasmodia, etc. From spores and/or zygotes of these organisms, daughter independent unicellular organisms are formed, which then gather into a new colony, or the zygote gives rise to a multinuclear plasmodium (see, for example, Novozhilov and Gudkov 2000: 417–443).

A similar situation occurs in the case of the formation of various specialized colonies (coenobia) of unicellular algae (for example, *Coelastrum* Nägeli, 1849, *Scenedesmus* Meyen, 1829, *Sphaerocystis* Chodat, 1897 and many others, especially among green and diatom algae), which are the result of secondary accretion or immersion in a common mucosal capsule of initially independent, self-feeding and reproducing cells. Inside each cell of the coenobium, small zoospores are again formed, which coalesce into a tiny daughter coenobium inside the mother cell, and then are released due to the rupture of the wall of this cell (Matvienko 1977: 271).

I also do not consider as multicellular organisms various multinucleated coenocytes (= somatella, cytoids, polycystids, etc.), known in some complexly organized ciliates, opalines, sporozoans, dinoflagellates, foraminifera and other protists. All these organisms do not meet the first reproductive criterion of multicellularity formulated above. The bodies of some “colonial” ciliates, for example, from the genus *Zoothamnium* Bory de St. Vincent, 1824, formed as a result of incomplete monocytic budding. Nevertheless, the resulting “colony” remains a de facto unicellular formation, within which there are no partitions, and all parts of which are connected by cytoplasmic strands caused by the so-called spasmonemes (Foster et al. 1978). There is no division into cells and inside multinucleated bodies (cenocytes) of parasitic dinoflagellates of the genus *Haplozoon* Dogiel, 1906 (see Angel et al. 2021), which were earlier erroneously identified as primary multicellular organisms (see, for example, Ivanov 1968).

Some difficulty can be caused by the application of the first reproductive criterion in relation to various cases of asexual reproduction at the initial stages of development of a multicellular organism. So, for example, in some cnidarians (Cnidaria) under experimental conditions, individual blastomeres retain the ability to give rise to independent embryos (Zakhvatkin 1949: 217). In a number of multicellular green algae (Chlorophyta) from the orders Ulotrichales, Sphaeropleales, Oedogoniales, and simply organized Charophyta s.l. from the order Coleochaetales the so-called “unicellular sporophyte” is preserved in the life cycle; it is a zygote, which is covered with a protective membrane and, after a dormant period, divides meiotically (and then mitotically), giving rise to 4–32 haploid zoospores (Belyakova et al. 2006b: 221, 267). In many red algae (Rhodophyta), the zygote gives rise to the so-called “gonimoblast filaments” (see more details below). In all these cases, no separate unicellular cycle of nutrition, development, and reproduction arises, since the mentioned zygotes are not independent organisms, and the products of their division obligatory grow into multicellular bodies.

Regular polyembryony, which occurs in a number of groups of highly developed animals and plants, is all the more not an example of unicellular reproduction, since it is realized on a multicellular basis (with the exception of random developmental anomalies in some individuals). First, a multicellular body of the embryo begins to form from a zygote or a parthenogenetic egg, and only then it is divided into several or many daughter embryos (Ivanova-Kazas 1977: 199–213, 1995: 480), i.e., in fact, we are talking about some kind of monocytic or polycytic budding (see more details below) in all studied examples of regular polyembryony. In higher plants, “polyembryony” is often understood not as the division of one embryo into several daughter ones, but as the appearance of many embryos and embryoids from different cells of the embryo sac, nucellus, and ovulum (Reproductive Systems 2000: 401).

A certain difficulty is also caused by the understanding of multicellularity in secondarily simplified parasitic animals – orthonectids (Orthonectida), in which one of the stages of the life cycle is a multinuclear “plasmodium”, capable of reproducing by monocyte budding. However, inside such a plasmodium, in addition to trophic nuclei, there are also generative nuclei with isolated sections of the cytoplasm, which are agametes (Malakhov 1990: 49; Slyusarev 2008). Thus, the body of these organisms is not a simple plasmodium, known in different protists, but a system of small cells located inside another, larger cell - a phenomenon known for a number of groups of animals and higher plants (see more details below).

The second reproductive criterion of multicellularity determines exactly how a multicellular body reproduces itself by the monocyte method of forming a daughter organism and allows us to divide all known ways of implementing obligate multicellularity into three fundamentally different variants.

The simplest and most archaic variant is protonemal multicellularity, in which a spore or zygote divides monotomically (by mitosis or simple cytokinesis), forming a single filament, a protonema (Fig. 1).

Monotomic division implies the obligatory growth of daughter cells after their division. As a result, a multicellular structure is formed from cells of approximately the same size, quite similar to the original cell or even exceeding its size. Such a single-row thread can then grow, branch many times, intertwine, forming a multilayer body (thallus). Protonemic multicellular organisms include the following groups:

1. Multicellular species of cyanobacteria (Cyanobacteria), actinobacteria (Actinobacteria), caryophane bacteria (Caryophanales) and some other prokaryotic groups. Cases of palintomy occurring in prokaryotes (for example, in the cyanobacteria *Gloeocapsa* Kützing, 1843, *Mycrocystis* Kützing, 1833, etc.) lead to the formation of independent daughter cells “nanocytes”, while multicellular bacterial thalli are formed from spores (akinetes) in a monotomic way (see, for example, illustrations in Kaplan-Levy et al. 2010).
2. Some genera of golden algae (Chrysophyceae), for example, *Hydrurus* Agardh, 1824, *Nematochrysis* Pascher, 1925, *Phaeodermatium* Hansgirg, 1889, etc.
3. Separate genera of yellow-green algae (Xanthophyceae), such as *Tribonema* Derbès et Solier, 1851, *Xanthonema* Silva, 1979, *Heteropedia* Pascher, 1939, *Heterococcus* Chodat, 1908, etc.
4. Some genera of pheotamniophic algae (Phaeothamniophyceae), for example, *Phaeothamnion* Lagerheim, 1884 and possibly *Sphaeridiothrix* Pascher et Vlk, 1943.
5. Isogamous and heterogamous genera of brown algae (Phaeophyceae), for example, from the orders Discosporangiales, Sphacelariales, Ectocarpales, etc., as well as the monotypic genus *Schizocladia* Henry et al., 2003, which the authors of this taxon propose to consider as an independent class Schizocladiophyceae, sister to brown algae.
6. Most multicellular red algae (Rhodophyta), with the exception of a number of highly developed genera (see below), in which an embryogenic variant of the development of bodies from carpospores and tetraspores is observed.
7. Obligate multicellular representatives of green algae (Chlorophyta s.l.) that meet the first reproductive criterion of multicellularity. For example, *Microthamnion* Nägeli, 1849 (Trebouxiophyceae: Microthamniales), *Schizogonium* Kützing, 1843, *Prasiola* Meneghini, 1838, *Raphidonema* Lagerheim, 1892 (Trebouxiophyceae: Prasiolales), *Protococcus* Agardh, 1824 (Chlorophyceae: Chlamidomonadales* ), some genera of Sphaeropleales*, most representatives of Ulvales, Ulotrichales, Trentepohliales, Chaetophorales, and Oedogoniales.
8. Obligate multicellular representatives of charophyta algae (Charophyta s.l.) from the classes Klebsormidiophyceae, Zygnematophyceae, and Coleochaetophyceae. To the contrary, highly organized charophyceous algae (class Charophyceae s.s.) develop according to the type of embryogenic multicellularity (see below).
9. The gametophytes of many genera of higher plants, especially bryophytes (Bryomorphae) and ferns (Pteridophyta), but in some cases also Lycopodiophyta, retain the simple protonemal character of spore germination. On the protonema, by budding, more complex bodies of gametophytes, differentiated into tissues and organs, can subsequently form. However, in other genera of the same plant groups, spores undergo palintomic/syntomic cleavage and develop according to the type of embryogenic multicellularity (see below).

**Figure 2.**
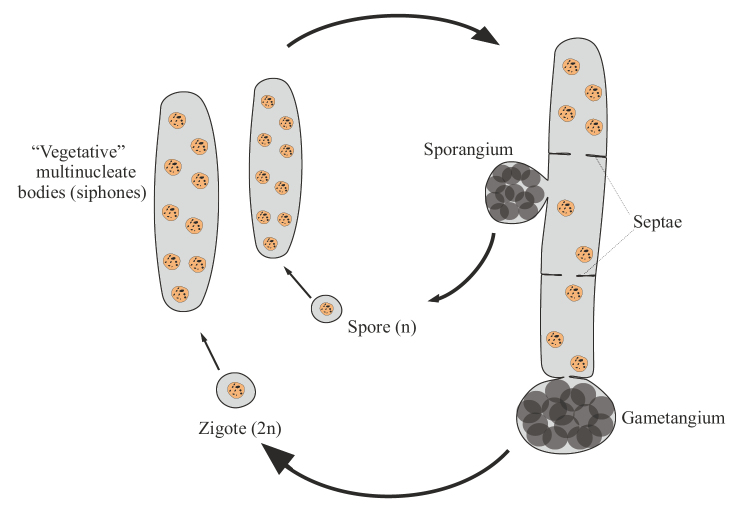
Generalized scheme of the life cycle in siphonoseptal multicellular organisms.

The second variant is siphonoseptal multicellularity (Fig. 2). Here, the zygote or spore initially undergoes multiple karyokinesises without division of the cytoplasm and forms a multinucleated cell, i.e. cenocyte. Further, this cell grows apically, sometimes reaching macroscopic dimensions of several tens of centimeters, and inside such a body, called the term “siphon”, regular or irregular partitions (septae) appear, dividing this siphon into multi-core compartments or clades (from the Greek “κλάδος” – a branch) with a different, less often the same, number of nuclei. Septae are formed by centripetal ingrowth of the membrane and cell wall into the inner cavity of the cell (Fritsch 1929; Egerod 1952; Enomoto and Hirose 1971; McDonald and Pickett-Heaps 1976; Liliaert et al. 2007; Okuda et al. 2016). This variant of body formation is well known in a number of genera of green algae of the order Siphonocladales and some other not closely related genera of green algae (see below). However, in fact, the same principle of the formation of a multinuclear thallus, divided into sections by septae, also takes place in various multicellular fungi and fungi-like organisms, including those that form septae only to separate sporangia and gametangia from a multinuclear hypha. The latter is typical, for example, for many oomycetes (Oomycota) and chytridiomycetes (Chytridiomycota). For this reason, I propose to understand siphonoseptality as a variant of multicellularity that arose independently in different groups of fungi and algae. A peculiar formation of irregular “septae” growing centripetally is also known during the formation of colonies in some mycobacteria (Dobrovolskaya 1974: 299).

Unfortunately, the ultrastructural and biochemical mechanisms of septa formation in multicellular algae, fungi, and, especially, prokaryotes, remain insufficiently studied, and the available knowledge is limited to single model objects (Barr and Gruneberg 2007; Seiler and Heilig 2019). In addition, in some siphonoclad algae (*Siphonocladus* Schmitz, 1879, *Dictyosphaeria* Decaisne, 1842, *Cladophoropsis* Børgesen, 1905, *Boodlea* Murray et De Toni, 1889, *Struvea* Sonder, 1845, and *Chamaedoris* Montagne, 1842), instead of the formation of septae, a special “segregative” division of body into separate parts occurs, and these parts then fuse again (Egerod 1952; McDonald and Pickett-Heaps 1976; Liliaert et al. 2007; Okuda et al. 2016). In fact, such bodies are not multicellular, but are just colonies of cenocytes, each of which, having separated, can give rise to a new organism.

It should be noted that the structure of the septate bodies of fungi and algae is not similar to the complicated construction of some protists (Protista), for example, gregarine (Gregarinea). In the latter, a single cell is sometimes divided into communicating parts by a “tangle of thin fibrils” (Simdyanov 2007: 50), while in parasitic dinoflagellates of the genus *Haplozoon* Dogiel, 1906, a single coenocyte is partially divided due to “alveolar vesicles” (Angel et al. 2021).

Siphonoseptal multicellularity is characteristic of the following groups:

1. A number of genera of Ulvophyceae green algae from the order Siphonocladales (for example, *Anadyomene* Lamouroux, 1812, *Cladophora* Kützing, 1843, *Valonia* Agardh, 1823, etc.), individual representatives of the related orders Dasycladales and Siphonales, in which septa are formed during the separation of rhizoids, sporangia and gametangia (for example, *Bryopsis* Lamouroux, 1809, *Derbesia* Solier, 1846, *Pseudobryopsis* Berthold, 1904, etc.), as well as the genus *Sphaeroplea* Agardh, 1824 from the order Sphaeropleales (see Fritsch, 1929).
2. Some genera of yellow-green algae (Xanthophyceae or Tribophyceae) from the order Vaucheriales. The multinuclear branching filaments of these algae usually lack septae, but their sporangia and gametangia are separated by septae.
3. Various groups of multicellular fungi and fungi-like organisms (as Oomycota, Chytridiomycota, etc.). In some fungi, for example, powdery mildew ascomycetes of the order Erysiphomycetes, there is a regular formation of septae with successive formation of mononuclear compartments of the hyphae (Belyakova et al. 2006a: 240). In many cases, especially during the formation of fungal “fruiting bodies”, false tissues are formed due to close fusion and even anastomoses between hyphae. This phenomenon is in many ways reminiscent of the secondary fusion of multinucleated cenocytes in siphonoclad algae, which are characterized by segregative division of the original cell.
4. It is possible that siphonoseptal multicellularity is also present in some ichthyosporids (Ichthyosporea), which are considered a group close to fungi and animals. At least some species of ichthyosporids form multinucleated thalli separated by septae, or such septae separate sporangia from the main “vegetative” body (Karpov 2011: 342–369). On the other hand, some ichthyosporid species have been suggested to have syntomic cell division (Suga & Ruiz-Trillo 2013). In general, ichthyosporids remain a poorly studied group, and the presence of a sexual process in them is assumed, but not proven.

**Figure 3.**
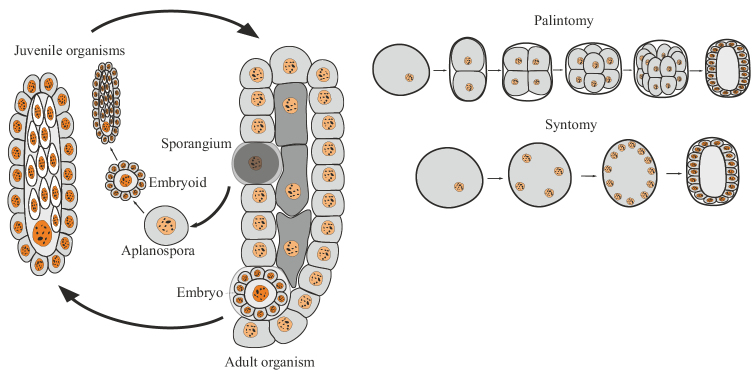
Generalized scheme of the life cycle and initial stages of development in embryogenic multicellular organisms.

Finally, the third and most complicated variant is embryogenic multicellularity (Fig. 3). It arises on the basis of obligate accumulative oogamy or accumulative aplanosporia, in which the gamete/spore exceeds in size (sometimes hundreds and even thousands of times (Ivanova-Kazas 1975: 39)) the original mother cells. As a result of palintomic or syntomic divisions, an embryo or embryoid is formed from an oogamete/spore (see below). Actually, only with this variant of reproduction for the first time in the evolution of living systems does the embryo appear as a biological phenomenon. In asexual monocytic reproduction, the analog of the oogamete is a large, immobile spore, the aplanospore, which gives rise to the embryoid. The term “embryoid” is widely and very ambiguously used in botany (less often in zoology) to refer to a variety of germ-like bodies arising from somatic cells (Reproductive Systems 2000: 334). I consider it expedient to understand by embryoids only cases of complete analogy with the sexual embryo: the emergence of the body from a single cell, enlarged in size, undergoing palintomic or syntomic divisions. The remaining cases of the emergence of daughter bodies from somatic cells I refer to budding.

The embryogenic variant of multicellularity is observed in the following organisms.

**Figure 4.**
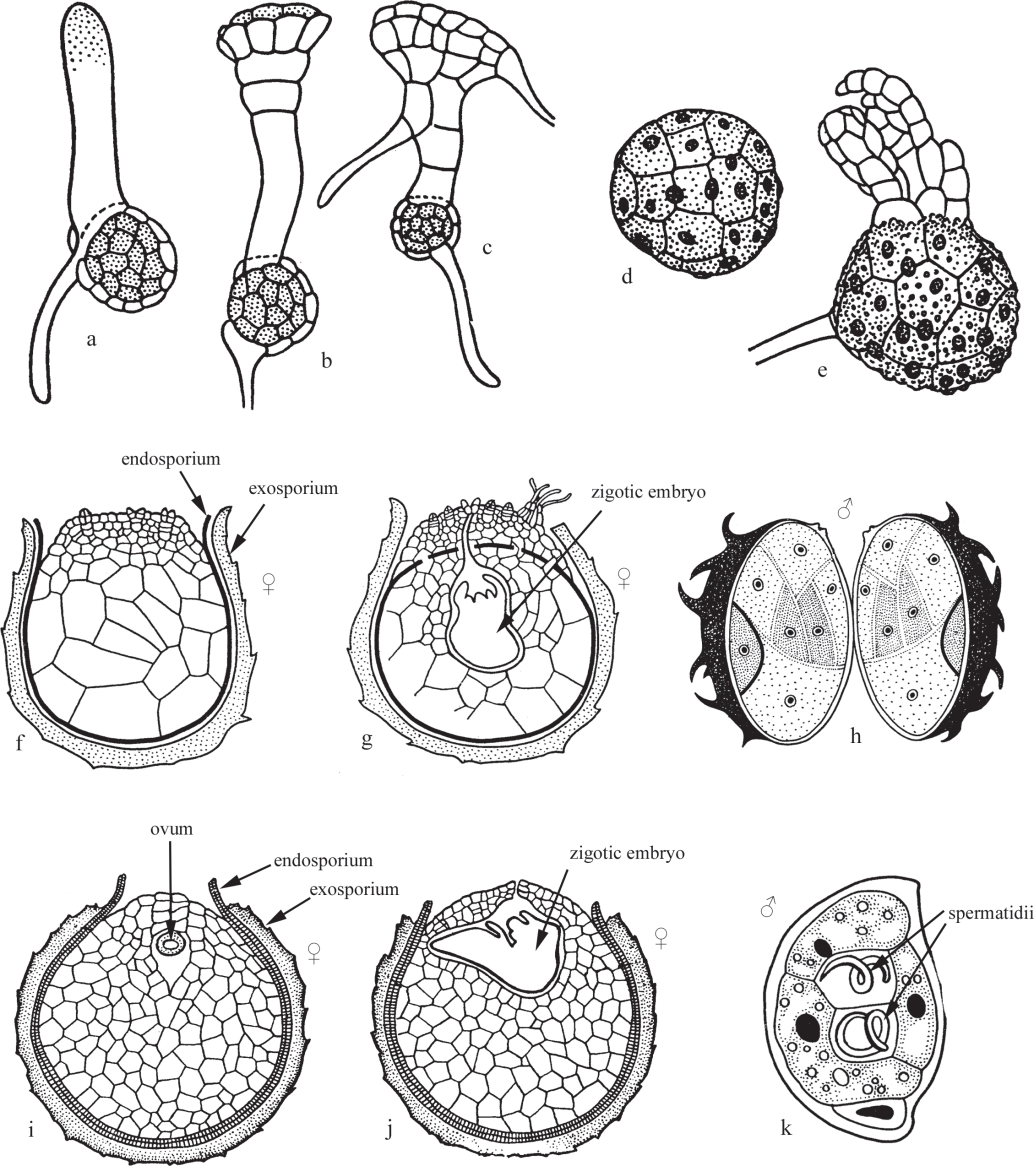
Embryoid gametophytes of higher plants. **a–c***Rebouliahemisphaerica* (Linnaeus, 1753) (Marchantiophyta) **d, e***Frullaniamuscicola* Stephani, 1894 (Marchantiophyta) **f–h***Selaginella* spp. (Lycopodiophyta); **i–k***Isoetes* sp. (Lycopodiophyta) **a–e** after Abramov and Abramova 1978, with changes **f–k** after Filin 1978, with changes.

1. All animals (Metazoa) as a holophyletic group that originally arose on the basis of embryogenic multicellularity.
2. Sporophytes of all higher plants (Embryophyta).
3. Gametophytes of a number of genera of higher plants (Bryomorphae, Lycopodiophyta, and Pteridophyta), in which spores undergo palintomic/syntomic fragmentation inside their shell, often still inside sporangia (Fig. 4). In bryophytes (Bryomorphae), such spores give rise to a multicellular embryoid, from which a more or less large gametophyte then grows (see review in Nehira 1983). Such an embryoid looks quite similar to the embryos arising from the zygote and giving rise to the sporophyte generation. In Lycopodiophyta, gametophytes are microscopic organisms, in most cases formed as a result of palintomic or syntomic spore cleavage, while in Selaginellopsida and Isoetopsida gametophytes do not leave the spore shell at all (Filin 1978; Timonin and Filin 2009: 181–221) (Fig. 4). Among ferns (Pteridophyta), palintomic/syntomic division of the spore is characteristic of heterosporous ferns, while homosporous ferns retain the protonemal character of gametophyte development (Nayar and Kaur 1971; Timonin and Filin 2009: 221–312).
4. Charophyceae s.s. in the traditional narrow sense.
5. Oogamous genera of brown algae (Phaeophyceae) from the orders Fucales, Desmarestiales, Dictyotales, Laminariales, Chordales, Tilopteridales, Sporochnales, etc. (see the summary table of such genera in Luthringer et al., 2014), characterized by complex differentiation of cells and tissues, like the sporophytes of higher plants. Some of these genera (for example, *Fucus* Linnaeus, 1753, *Sargassum* Agardh, 1820, etc.) have a diplontic life cycle with gametic meiosis. That is, the reduction in the number of chromosomes occurs during the formation of gametes, similar to how it takes place in the life cycle of animals; there is no haploid generation in such a cycle. Other genera (for example, *Dictyota* Lamouroux, 1809, *Padina* Adanson, 1763, etc.) demonstrate a haplodiplontic life cycle with isomorphic generations, i.e. gametophytes are morpho-anatomically quite similar to sporophytes. In the third group of genera (for example, *Himantothallus* Scottsberg, 1907, *Desmarestia* Lamouroux, 1813, *Laminaria* Lamouroux, 1813, etc.), heteromorphism of generations is observed (Petrov 1977: 143–192; Luthringer et al. 2014). In these cases, sporophytes usually have an embryogenic origin, while strongly reduced filamentous gametophytes develop from a protonema or even represent a single cell. In addition, examples of irregular alternation of haploid and diploid generations, parthenogenetic germination of gametes and the formation of microscopic protonemal sporophytes (“plethysmothallus”) are known in brown algae, capable of producing not only spores, but also directly give rise to a macroscopic thallus (Petrov 1977: 143–192). All this confusing picture of the reproductive strategies of Phaeophyceae probably indicates the multiple independent origin of oogamy and embryogenic multicellularity in them during the haploid and/or diploid phases of the life cycle. Some authors (Heesch et al. 2021) make unexpected suggestions about secondary transitions from oogamy to heterogamy and isogamy in brown algae. However, it should be noted that these hypotheses are based solely on the belief in the infallibility and universality of molecular statistical cladism as a method of phylogenetic reconstructions.

It is interesting that in a number of works on various genera of brown algae, for example, in the articles by Nanda (1993), Edwards (2000), Kawai et al. (2001), and Bogaert et al. (2017), the initial stages of development of these algae are directly called embryonic, i.e. the similarity of the division of their zygotes with the embryonic development of higher plants and animals was noted.

1. Some genera of red algae (Rhodophyta). In species some highly developed genera, for example, *Corallina* Linnaeus, 1758, *Dumontia* Lamouroux, 1813, *Jania* Lamouroux, 1812, *Amphiroa* Lamouroux, 1812, *Gracilaria* Greville, 1830, etc., palintomic divisions of aplanospores (“tetraspores” and “carpospores”) is observed with subsequent formation a kind of hemispherical multicellular disk (Chemin 1937; Jones and Moorjani 1973; Michetti et al. 2013; Wai 2018; etc.). This structure is quite consistent in origin with the embryoids formed during asexual reproduction in other groups of plant organisms. Some authors (Chemin 1937: 369) have even compared this development with the formation of the embryonic morula of animals.
2. Oogamous species of green algae of the genus *Volvox* Linnaeus, 1758, s.l., with differentiation of cells connected by plasmodesmata. The embryogenic origin of the multicellularity of oogamous *Volvox* spp. is well known in the literature and described in detail in many works, for example, by Zakhvatkin (1949: 220–232). A review of more recent data can be found, for example, in Desnitsky (2018).

From the above list of organisms, it can be seen that embryogenic multicellularity did not arise on the basis of prokaryotic cells. This fact, of course, is not accidental and is probably due to the fact that prokaryotic cells are not capable of providing effective intercellular transport of substances and, accordingly, of the formation of differentiated tissues. As a result, prokaryotes do not have examples of the embryonic development required for initial cell differentiation. Moreover, due to the absence of the endoplasmic reticulum, the transport of substances within prokaryotic cells is limited by the possibilities of diffusion, which imposes significant restrictions on cell size. Large sizes (sometimes up to 0.75 mm in diameter) of cells in some prokaryotes, for example, in the bacterium *Triomargaritanamibiensis*Schulz et al., 1999, are explained by the fact that the entire central part of such cells is occupied by vacuoles, while the cytoplasm forms only a thin peripheral layer (Schulz et al. 1999).

## ﻿Monocytic reproduction of protonemal multicellular organisms

Monocytic bisexual reproduction in protonemal multicellular organisms can proceed according to the type of isogamy, heterogamy, oogamy, or analogs of oogamy, whereas asexual monocytic reproduction can proceed according to the type of zoosporia or aplanosporia. Evolutionary models for the emergence of gamete diversity (anisogamy) from the initial isogamous sexual process have been repeatedly proposed in the specialized literature (Parker at al. 1972; Bell 1978; Bulmer and Parker 2002; Umen and Coelho 2019, etc.; see also the review by Blute 2012) and therefore there is no need to dwell on the discussion of this issue here. In general, there is no doubt that the appearance of anisogamy, with rare exceptions, directly correlates with an increase in the complexity of the body of an organism and, in particular, with the appearance of multicellularity (Bell 1978).

The various evolutionary transformations within the broadly understood oogamy deserve more detailed consideration, since, as will be shown below, oogamy is a necessary prerequisite for the transition to complex forms of multicellularity. The oogamous sexual process (or its analogues) in protonemal multicellularity is still carried out in an extremely achaic way, since in this case the oogamete (with rare exceptions) does not accumulate nutrients for further development, but remains comparable in volume to usual somatic cells or even turns out to be significantly smaller than the latter. As a result of this, the further development of the parthenogenetic or fertilized oogamete (zygote) inevitably occurs through monotomic germination, i.e. successive division and growth of daughter cells forming a filamentous structure (protonema).

**Figure 5.**
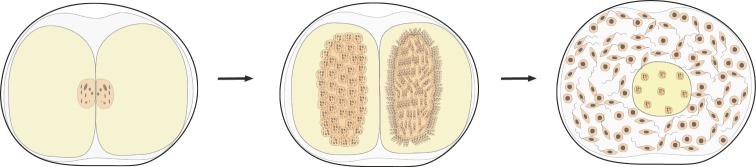
Formation of syzygy and copulation in the gregarine *Stylocephaluslongicollis* (Stein, 1848).

It should be noted that examples of archaic oogamy are already found in unicellular and unicellular-colonial organisms. Thus, some genera of colonial diatoms (Diatomophyceae), for example, the so-called centric diatoms (orders Thalassiosirales, Coscinodiscales, Melosirales, Chaetocerotales) and pennate diatoms of the genus *Rhabdonema* Kützing, 1844, demonstrate oogamy, in which germ cells are smaller than somatic ones (Belyakova et al. 2006b: 85–93; Kaszmarska et al. 2013; Davidovich 2019: 31, 62). A similar archaic oogamy is known in some unicellular Trebouxiophyceae algae (Gonzalves and Mehra 1959). In most of the studied species of gregarine (Gregarinea), during sexual reproduction, two parental haploid cells unite, forming the so-called syzygy, and become covered by a common membrane (Fig. 5). Inside the shell of the syzygy, each parent cell divides by syntomy (schizogony) and forms gametes. The latter can be the same in size and functionality (isogamy) or differ significantly (anisogamy). In different genera of gregarines, immobile “female” gametes can, at the same time, be larger or smaller than mobile “male” gametes with flagella (Zakhvatkin 1949: 197; Grassé 1953; Simdyanov 2007: 26, 52–61). In most cases, the resulting gametes are many times smaller than the original parental cells, or slightly smaller (when a single zygote is formed inside the syzygy), but never exceed them in size. The fusion of gametes occurs inside the shell of the syzygy. Each resulting zygote is surrounded by its own protective shell and becomes an “oocyst”. Subsequently, the “oocyst” undergoes two meiotic divisions, and the resulting haploid cells give rise to a new generation of unicellular or polycystid gregarines (Simdyanov 2007: 33, 50).

In the related group of coccidia (Coccidea), the oogamete is formed directly from the haploid parent cell (merozoite) without division of the latter, and biflagellated (rarely non-flagellated) male gametes arise as a result of syntomic division of the merozoite. The possibility of fusion of gametes in this case is achieved by the fact that the parent cells are in close proximity to each other inside the body of the host organism. Meiosis in the life cycle of coccidia, as in gregarines, occurs in the “oocyst” formed from the zygote (Beyer 2007: 149–248).

**Figure 6.**
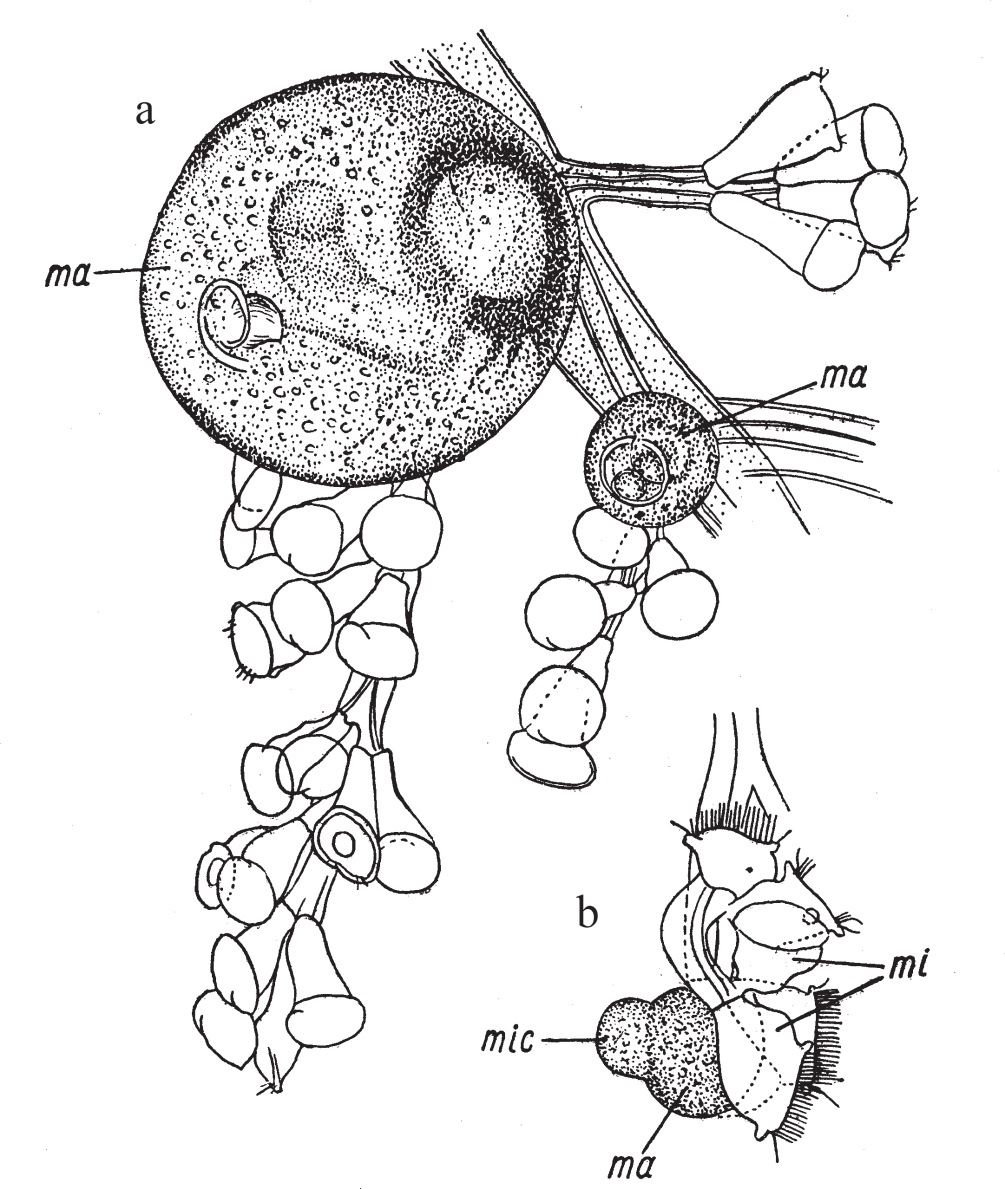
Analogy of the oogamous sexual process in the ciliate *Zoothamniumarbuscula* Ehrenberg, 1839 **a** colony with macrozooids (ma) **b** conjugation (mac – macroconjugant, mic – microconjugant, mi – microzooid). After Fursenko 1924 and Ivanov 1968.

Some highly developed ciliates that form “colonies” by incomplete budding (Fursenko 1924; Ivanov 1968: 30–31) demonstrate a kind of analogue of oogamy, in which the “macrogamete” (macrozooid) remains motionless, and the mobile small “microgamete” (microzooid) swims up and carries out “fertilization” (Fig. 6).

Relatively few examples of archaic oogamy (without an increase in the size of the gamete) are known among protonemal multicellular organisms. For example, such oogamy has been well studied in green algae of the genus *Prasiola* Meneghini, 1838 (Trebouxiophyceae: Prasiolales). In the upper part of their multicellular diploid thallus, meiotic divisions occur and biflagellated spermatozoa and non-flagellated oogametes (ova) are formed. Female gametes are about twice as large as male, but smaller than the original diploid cells of thallus. They are released due to the destruction (“dissolution”) of the lower cell walls of thallus and end up in a bubble-like space bounded by the persistent outer common shell of the thallus (“persisting bladder-like coating lamella”). At the same time, hundreds or even thousands of heterosexual gametes are released into this space and fertilization occurs. A protonema grows from the zygote, and a new diploid thallus grows from it (Friedmann 1959; Cole and Akintobi 1963). Thus, there is hermaphroditism and self-fertilization in a closed space, which resembles the corresponding processes in various intracavitary parasitic organisms.

**Figure 7.**
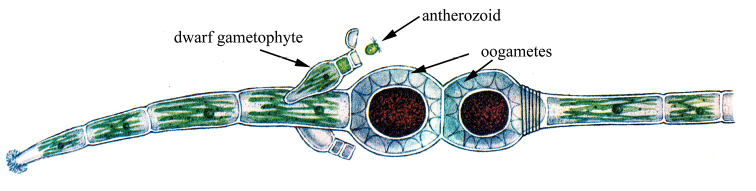
Accumulative oogamy in protonemal multicellularity in *Oedogoniumstellatum* Wittrock ex Hirn, 1900 (after Vinogradova 1977b, with modifications).

Even rarer in protonemal multicellular organisms, accumulative oogamy occurs, in which an increase in the volume of the egg takes place in comparison with the cells of the “vegetative” body that preceded it (Fig. 7). This variant is known in a number of multicellular green algae (Chlorophyta) from the orders Ulotrichales, Oedogoniales and in simply organized members of charophyta algae (Charophyta s.l.) of the order Coleochaetales. In their life cycle, the so-called “unicellular sporophyte” is preserved, which is a zygote, covered with a protective membrane and, after a dormant period, divides meiotically and then mitotically, giving rise to 4–32 haploid zoospores (Vinogradova 1977b: 282, 285; Belyakova et al. 2006b: 221, 267). In Oedogoniales, special “androspores” settling on the oogonium or cells adjacent to it form peculiar dwarf gametophytes – “nanandria”, the upper cells of which function as antheridia (Vinogradova 1977b: 293; Belyakova et al. 2006b: 254) (Fig. 7). A similar process is observed in *Cylindrocapsopsis* Iyengar, 1957 (Chlorophyceae: Sphaeropleales) (Vinogradova, 1977b: 294).

**Figure 8.**
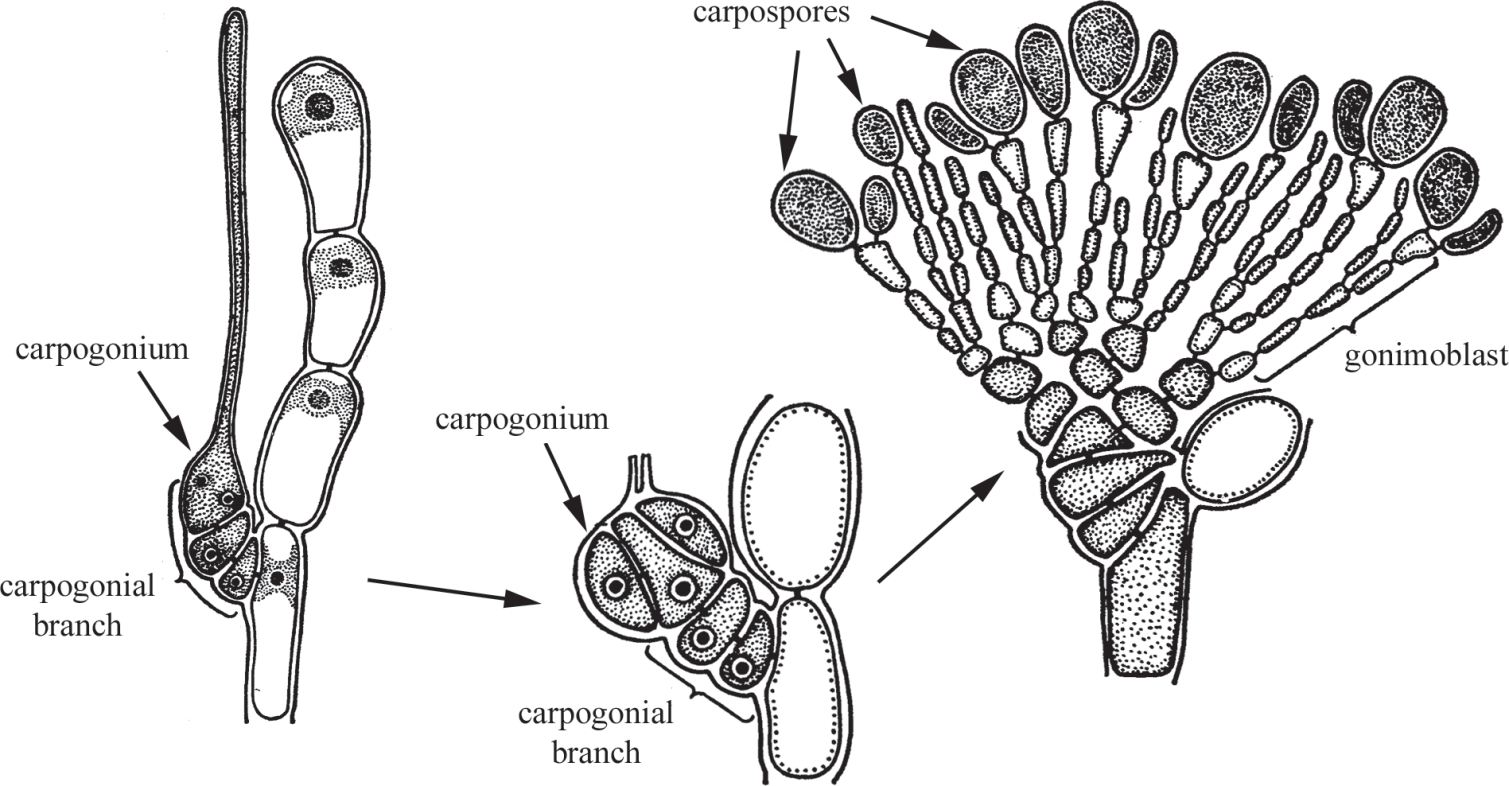
Scheme of development of the “carposporophyte generation” of floridian red algae (Rhodophyta: Florideophyceae). After Vinogradova 1977a, with changes.

A peculiar analogy of archaic oogamy among protonemal organisms occurs in red algae (Rhodophyta) (Fig. 8). Their male gametes (sperms) are devoid of flagella and are passively transferred to the female genital organs (carpogons). There is no female gamete as such. The male gamete fuses with the carpogon nucleus. The fusion nucleus then grows into a diploid gonimoblast (“carposoporophyte”). Carposporangia producing spores are formed on the gonimoblast. These spores form the “second diploid generation – the tetrasporophyte” (Vinogradova 1977a).

The increase in the number of individuals in protonema-multicellular organisms occurs mainly during the production of spores. In fact, the spore in archaic organisms is quite homologous to the unfertilized gamete, which was convincingly shown, for example, in the fundamental work of Zakhvatkin (1949; 1956). In many algae, unfertilized gametes, including flagellar ones, can develop into new thalli – see, for example, Smith 1947; Belyakova et al. 2006b: 225–226, 234.

It is well known that, similar to the evolutionary transition from small mobile gametes to large immobile gametes, in various groups of organisms there is a transition from small zoospores to immobile aplanospores, which in many cases do not exceed ordinary somatic cells in volume, but in a number of organisms they accumulate nutrients and increase significantly in size. At the same time, the production of a large number of small spores is typical for most protonemal multicellular organisms. Particularly impressive examples are demonstrated by some genera of red algae: each sporophyte produces about 12 million carpospores, and one tetrasporophyte produces 100 million tetraspores (Vinogradova 1977a: 212).

**Figure 9.**
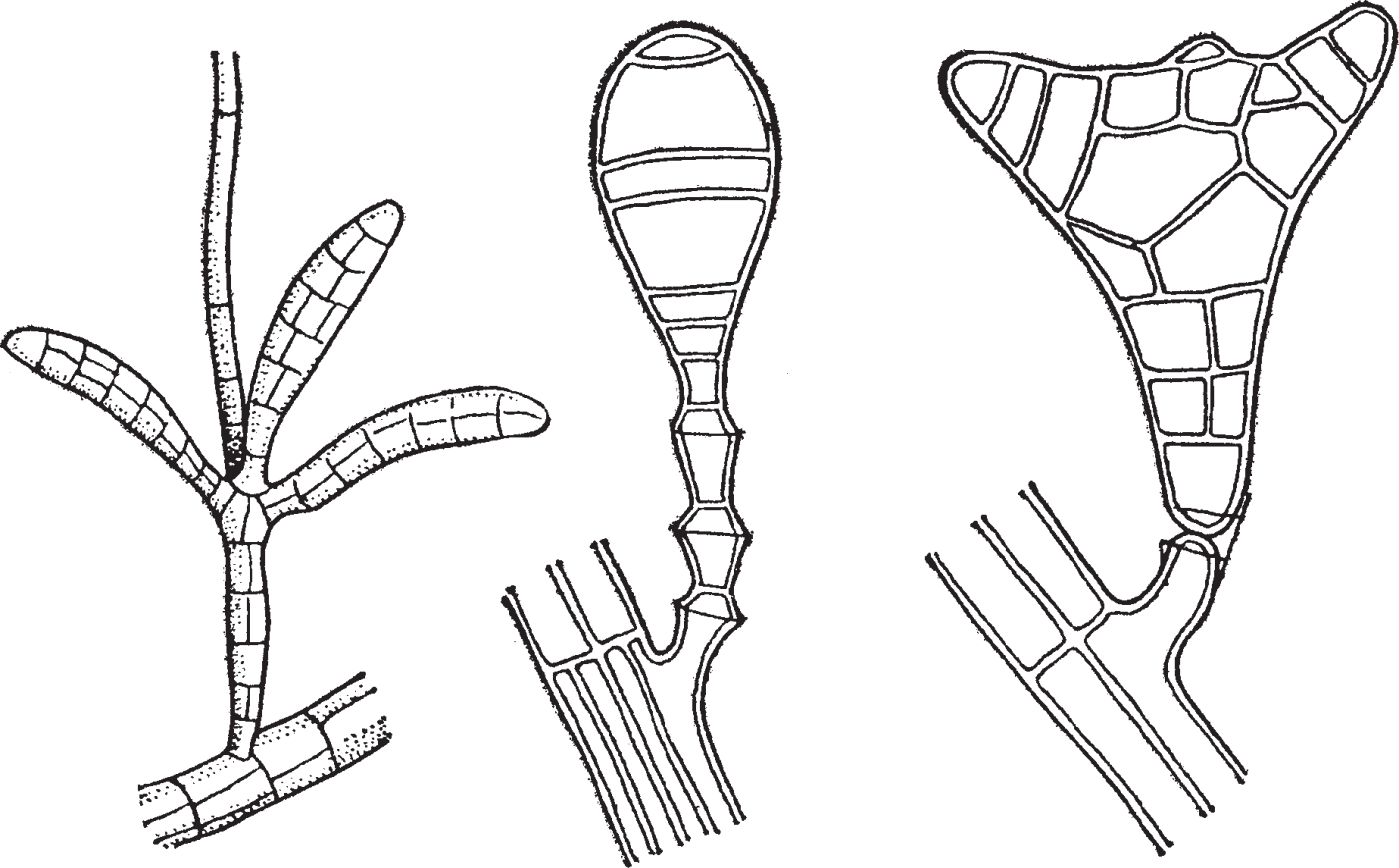
Monocytic budding in protonemal multicellularity, on the example of species of the genus *Sphacelaria* Lyngbye, 1818 (after Petrov 1977, with changes).

Homologous to the process of sporulation can be considered monocytic budding of protonemal multicellular organisms. Thus, in one of the isogamous genera of brown algae, *Sphacelaria* Lyngbye, 1818, vegetative reproduction is carried out by multicellular structures formed at the ends of branches (Petrov 1977: 162). Thus, each such structure arises from a single apical cell and is similar to a multicellular spore that began its development while still on the mother’s body (Fig. 9). Similarly, monocytic brood buds are formed in the gametophytes of some ferns (Gladkova 1978: 222–223).

## ﻿Monocytic reproduction of siphonoseptal multicellular organisms

The sexual process is predominantly isogamous or heterogamous. However, in some groups, archaic oogamy occurs (without an increase in the size of the eggs in comparison with the original cells of the mother’s body), analogues of oogamy, or somatogamy (fusion of two somatic cells).

**Figure 10.**
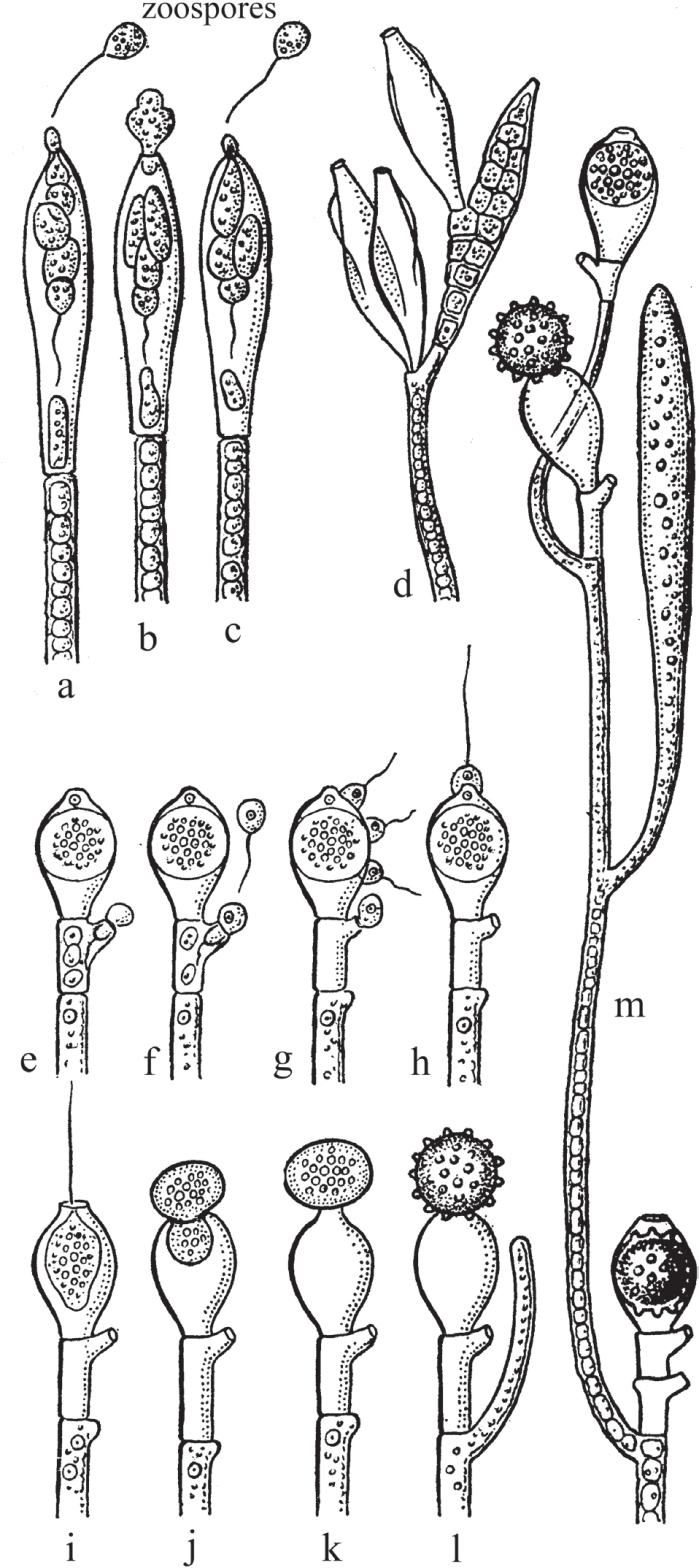
Asexual and sexual reproduction in siphonoseptal multicellularity on the example of *Monoblepharis* spp. **a–c** asexual reproduction by zoospores **d** branching of zoosporangia **e–l** successive stages of the sexual process **m** section of the siphon-septal body with genital organs and zygotes (after Sparrow 1933 and Sizova 1976, with changes).

Thus, in Chytridiomycota of the order Monoblepharidales, the multinuclear mycelium usually does not contain septa, but zoosporangia, oogonia, and antheridia are separated from the body by septae (Belyakova et al. 2006a: 157). Each oogonium produces one or more small ova (Fig. 10). Uniflagellated spermatozoa fertilize the egg inside the oogonium. The zygotes retain amoeboid movement, or they can move at the expense of one flagellum left from the fusion with the spermatozoon. After leaving the oogonium or inside it, the zygote is covered with protective layers and is at rest for some time. Subsequently, a multinucleated hypha develops from such a zygote (Sparrow 1933; Sizova 1976: 32–34).

In some genera of yellow-green algae (Xanthophyceae or Tribophyceae) from the order Vaucheriales, multinuclear branching siphons are usually devoid of septae, but sporangia and gametangia are separated by septae. In *Vaucheria* de Candolle, 1801 and *Pseudodichotomosiphon* Yamada, 1934, a single small egg cell is formed in the spherical oogonium, while numerous biflagellated spermatozoa are formed in the antheridia. The sperm enters the oogonium through a pore in the membrane. The diploid zygote, after a dormant period, germinates into a new thallus (Kobara and Chihara 1984; Belyakova et al. 2006b: 109–110).

In green algae of the genus *Sphaeroplea* Agardh, 1824 (Sphaeropleales), multinuclear siphonal bodies are separated by centripetally formed septae, but no special organs of sexual reproduction are formed. Small eggs and spermatozoa are formed in any of the segments of the body (Fritsch 1929). Fertilization is internal. The zygotes dress in dense shells and leave the mother thread only after the destruction of the latter. During the germination of the zygote, 4 zoospores are formed, each of which then gives rise to a new siphonoseptal plant (Vinogradova 1977b: 294).

An analogue of archaic oogamy in siphonoseptal multicellular organisms can be considered sexual reproduction of multicellular ascomycetes (Ascomycota), basidiomycetes (Basidiomycota), oomycetes (Oomycota) and some others. True oogamy is completely absent in these organisms. Instead, there are different variants of fusion of hyphae segments that function as unicellular gametangia, as well as the formation of male gametes (spermatia) in the absence of female ones, or vice versa (Belyakova et al. 2006a: 78).

Asexual monocytic reproduction of siphonoseptal multicellular organisms is usually carried out by small unicellular zoospores or immobile, including multicellular, aplanospores (ascospores, basidiospores, etc.). The formation of unicellular or multicellular conidia in many groups of fungi is considered a special variant of spore formation (Belyakova et al. 2006a: 77).

## ﻿Monocytic reproduction of embryogenic multicellular organisms

Monocytic reproduction of embryogenic organisms in all known cases is associated either with accumulative oogamy (during the sexual process or parthenogenesis), or with accumulative aplanosporia (during asexual reproduction). In both cases, a new organism begins to develop from one immobile cell, which is larger than the usual somatic cells of the organism. The division of such a cell occurs according to the type of palintomy, i.e. rapidly recurring karyokinesis and cytokinesis, without a period of growth of daughter cells, or by the type of syntomy, i.e. rapidly recurring karyokinesis followed by simultaneous division of all cytoplasm of the mother cell into numerous compartments (Fig. 3). As a result of such divisions, a special phase of ontogenesis appears — the embryo, as a fundamentally new biological phenomenon or an analogue of the embryo (embryoid), when it comes to development from a spore. Embryos (embryoids), unlike seedlings of protonemal and siphonoseptal multicellular organisms, are not capable of independent life; they are completely dependent on the supply of nutrients from the mother’s body or on the reserves of substances accumulated by the mother in the form of a “yolk” inside the egg. Thus, the following definitions of terms can be proposed:

**Embryo** — the initial stage of development of embryogenic multicellular organisms, from the first division of the zygote (or parthenogenetic egg) to the beginning of independent life (exit from the shell of the zygote (egg) or separation from the mother’s organism).

**Embryoid** — an analogue (in some cases also a homologue) of an embryo, the initial stage of development of embryogenic multicellular organisms during asexual monocytic reproduction, from the first divisions of the original cell (spore or spore-like cell) to the beginning of independent life.

Probably the most simple embryogenesis is saved in Charophyceae algae. Their zygote (“oospore”), while still inside its shell, undergoes syntomic divisions of meiosis, resulting in the formation of a four-nuclear cell. One of these haploid nuclei is separated by a septum, undergoes another palintomic division, and gives rise to root and stem cells. The remaining trinuclear cell performs the function of storing nutrients (Gollerbach 1977: 347). Thus, the embryonic stage is represented here by only a few cells. After reaching this stage, the zygote shell opens and intensive postembryonic growth of the root and stem begins in a monotomic way.

Oogamy and embryogenesis are most complex in animals (Metazoa). In them, unlike plant and fungal organisms, the process of female meiosis is in the nature of unequal division, resulting in the formation of one egg and several reductional (polar) bodies. The evolutionary meaning of this phenomenon is not clear, since it is not known in other embryogenic multicellular organisms. It is worth mentioning that in some animals, for reasons that are not entirely clear, male meiosis is also unequal (Hodgson 1997; Swallow and Wilkinson 2002; Gavrilov-Zimin et al. 2015; Gavrilov-Zimin 2018: 25–33).

In addition, a special variant of embryogenesis “a cell within a cell”, known for a number of secondarily simplified parasitic animals (myxosoporidia, cnidarians of the genus *Polypodium* Ussov, 1885, orthonectids, dicyemids), as well as for angiosperms (Magnoliopsida) looks unclear in terms of evolutionary meaning and causes of occurrence.

For a century and a half, myxosporea (Mixozoa) were considered by most zoologists as one of the groups of protists (Pugachev, Podlipaev 2007: 1045–1080), although the hypothesis of their belonging to multicellular animals (Metazoa) was first put forward as early as 1899 (Štolc 1899). In recent years, on the contrary, the point of view has become generally accepted that myxosporea are descendants of cnidarians (Cnidaria), greatly simplified due to parasitism. As arguments for the multicellularity of myxosporea, their structural and biochemical features, as well as comparison of nucleotide sequences of genes are given (Siddall et al. 1995; Foox and Siddall 2015). The reproductive features of myxosporea and the initial stages of their ontogeny, at first glance, seem unique and mysterious (see reviews: Feist et al. 2015; Gruhl and Okamura 2015). However, for all its aberration, the reproduction of myxosporea fundamentally corresponds to the complex embryogenic reproduction of multicellular organisms: 1) during meiosis, non-flagellated haploid gametes are formed, which can be considered eggs, since meiosis proceeds according to the female type, with the formation of polar bodies; 2) diploidy is restored parthenogenetically, although the details of this process remain largely unclear; 3) the parthenogenetic zygote splits palintomically, like the zygote of embryogenic multicellular organisms, although without the usual stages of blastulation and gastrulation; 4) as a result a multicellular (with cell differentiation) dispersal stage of the life cycle, called “actinospore” is formed; 5) inside the body of this dispersal stage, the so-called “sporoplasms” are formed asexually, giving rise to asexual generations.

In an even more aberrant, but still insufficiently studied way, the reproductive system functions in another parasitic representative of the coelenterates, *Polypodiumhydriforme* Ussov, 1885 (Cnidaria: Polypodiozoa). The sexual generation of this species is a free-swimming, dioecious freshwater jellyfish. Their female gonads are appeared during ontogenesis, but do not function. The male gonads produce non-flagellate, binuclear gametes that inexplicably enter the oocytes of the host organism (fish). Further, these gametes, without fertilization, proceed to unequal cleavage, as a result of which a kind of morula is formed, which is placed inside a large polyploid cell called a trophamnion. Embryogenesis lasts several years, in accordance with the development of the host’s oocytes, and ends with the formation of a larva that looks like an inside-out planula. Numerous “buds” are formed on the body of this larva and the whole organism takes the form of a stolon. Shortly before host spawning, the stolon inside the oocyte turns inside out and acquires the normal position of the cell layers for the coelenterates. The release of stolons from the eggs of the host occurs in the reproductive ducts of the fish. After entering the water, the polypodium stolon undergoes fragmentation with the formation of daughter medusoid forms (Raikova 1994).

**Figure 11.**
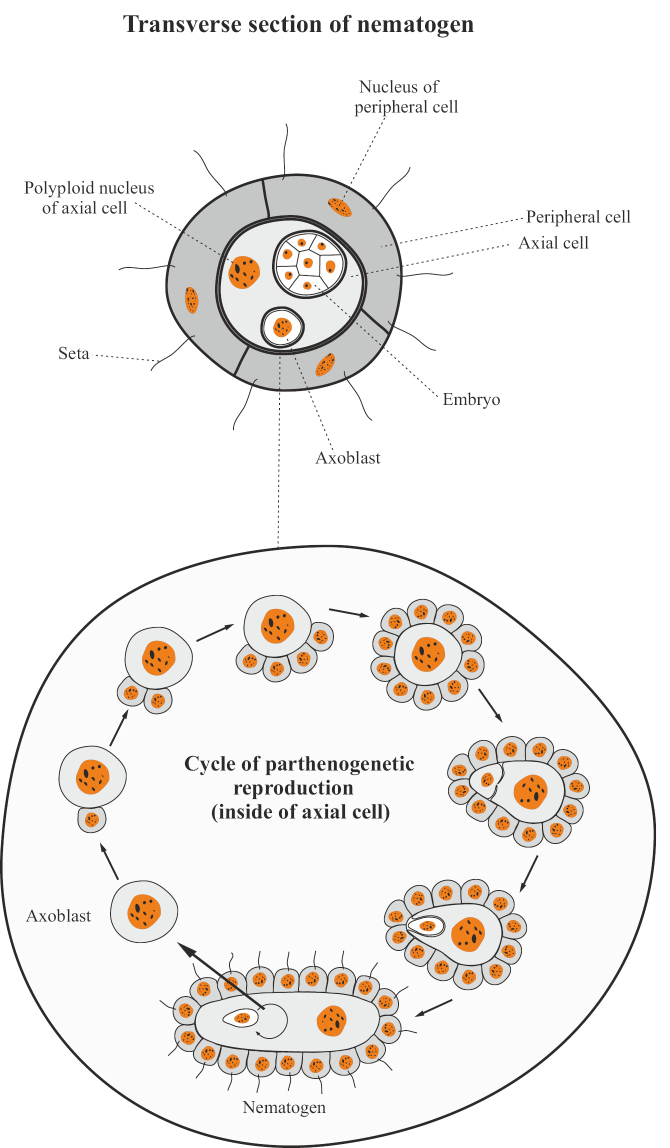
The structure of the nematogen and the cycle of parthenogenetic reproduction of Dicyemida.

**Figure 12.**
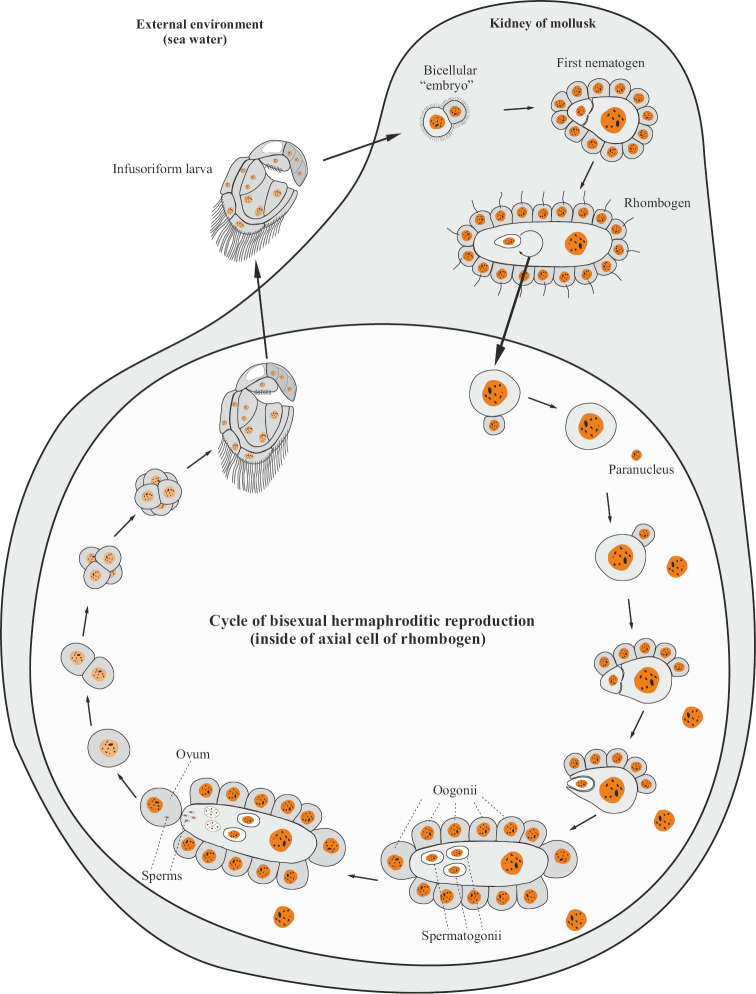
The cycle of sexual hermaphrodite reproduction of Dicyemida.

The body of dicyemides (Dicyemida) is arranged in an extremely simplified way, consists of only 8–40 cells and does not have any tissues, organs and intercellular cavities. The total number of cells is determined and characteristic of each species. The body of adult worm-like stages (nematogen and rhombogen) is formed by one layer of integumentary (somatic) ciliated cells and one (rarely several) large internal axial (generative) cell (Fig. 11) with a polyploid nucleus. In the internal cytoplasmic chambers of this cell, smaller cells are located — axoblasts, which give rise to individuals of the next generations (McConnaughey 1951; McConnaughey and McConnaughey 1954; Ivanova-Kazas 1975; Malakhov 1990; Furuya and Tsuneki 2003; Furuya et al. 2003; Noto et al. 2003). Reproduction is carried out by parthenogenetic and bisexual methods. During parthenogenesis, the axoblast undergoes irregular mitotic divisions such that the larger cell (macromere) becomes the new axial cell, and the micromere continues to divide, resulting in the formation of the next generation nematogen cover layer. Then the macromere stretches out and undergoes another uneven division; the smaller of the two daughter cells becomes a new axoblast and invades the cytoplasm of the larger cell. After these processes, the young nematogen leaves the parent individual, squeezing between its cells. In different species of dicyemides, from one or two to more than a hundred daughter nematogens can simultaneously develop in the axial cell (McConnaughey 1951; McConnaughey and McConnaughey 1954; Ivanova-Kazas 1975; Malakhov 1990; Furuya and Tsuneki 2003; Furuya et al. 2003). Thus, in dicyemids, the initial stage of reproduction is associated with a single cell (axoblast), which can be called an agamete or pseudogamete. In this case, one can speak of a special variant of ameiotic parthenogenesis, in which not only fertilization, but also reductional division of oocytes is absent (see Ivanova-Kazas 1975: 100). An adult dicyemid that performs sexual reproduction is called a rhombogen. Morpho-anatomically, this individual does not differ much from a nematogen, but in its axial cell, axoblasts form hermaphrodite “gonads”, called infusorigens. In this case, after the first unequal division of the axoblast, the micromere separates from the macromere, loses its cytoplasm and remains in the axial cell of the rhombogen in the form of a free nucleus called the paranucleus (Fig. 12). As a result of the accumulation of paranuclei originating from many embryos, the axial cell of the rhombogen gradually becomes multinucleated. As a result of subsequent unequal divisions of the macromere, micromeres are again separated from it, which give rise to oogonia and spermatogonia, and the original macromere itself becomes an axial cell of the infusorigen. Oogonia are located along the periphery, and spermatogonia are inside the axial cell as a result of the invagination of one of the micromeres into its cytoplasm and its subsequent divisions. Spermatogonial cells, after passing through meiotic divisions, lose most of their cytoplasm and form non-flagellate sperms from them. These sperms penetrate into the oocytes located near the axial cell and fertilize them. From the zygote, a mobile larval stage is formed — infusoriform, which leaves the maternal rhombogen, and then the kidney of the host organism (mollusk) and enters the external aquatic environment (McConnaughey 1951; McConnaughey and McConnaughey 1954; Ivanova-Kazas 1975; Malakhov 1990; Furuya and Tsuneki 2003; Furuya et al. 2003). The way infusoriform penetrates into a new host mollusk remains still insufficiently studied, but it is assumed that the so-called “urn cells” of infusoriform give rise to a two-cell “embryo” that grows into a founder nematogen, and that, in turn, produces new nematogens or rhombogens. From the above information, it becomes clear that the development of new nematogens, rhombogens and infusoriform larvae of dicyemides occurs entirely within the maternal organism, from which they receive the necessary nutrition. Thus, dicyemides as a whole, as a taxonomic group, should be considered viviparous organisms.

**Figure 13.**
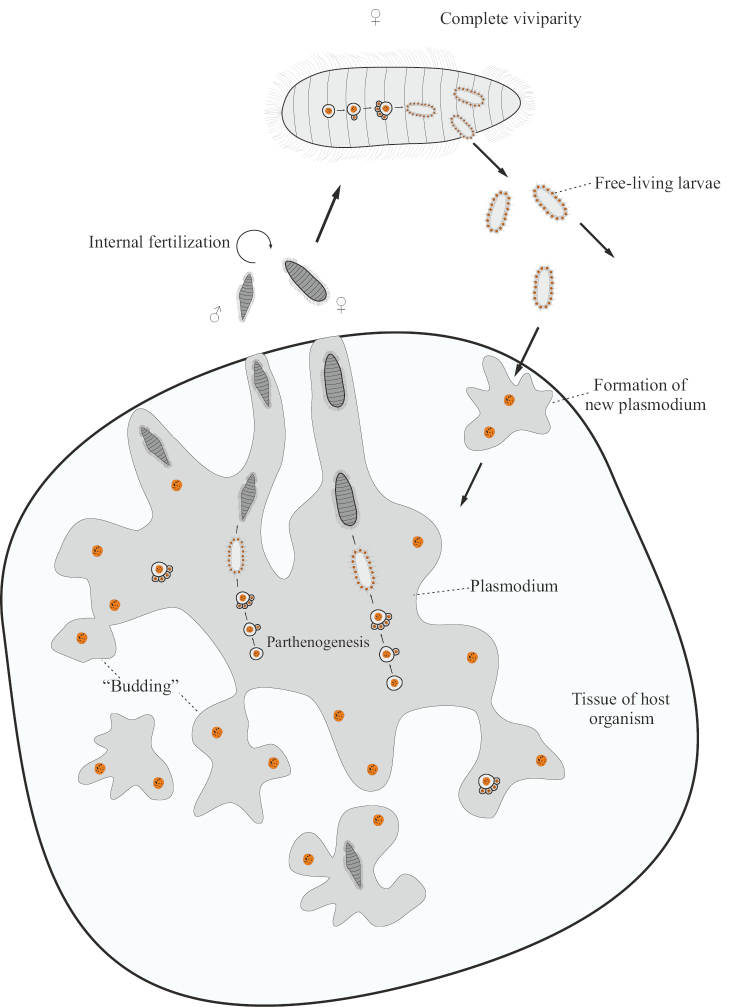
Generalized scheme of the life cycle of Orthonectida.

In orthonectids (Orthonectida), the main stage of the life cycle (Fig. 13) is a multinuclear plasmodium located in the tissues of the host organism. Plasmodium does not have a definite shape, and daughter plasmodia can be formed from different parts of its surface by simple budding. Inside the plasmodia, in addition to the trophic nuclei, there are generative nuclei with isolated sections of the cytoplasm, which are agametes. These agametes without fertilization undergo uneven fragmentation with subsequent gastrulation by the type of delamination (Ivanova-Kazas 1975: 92; Malakhov 1990: 49) and the formation of adults, bypassing the larval stage. As a result of this kind of parthenogenesis, males and females of the next (bisexual generation) are formed inside the plasmodia, and in different species, individuals of both sexes can form inside one plasmodium or in different plasmodia. Sexual individuals of orthonectids are not capable of self-feeding, but have significant mobility due to numerous setae covering the body. Eggs and spermatozoa are formed in sexual individuals even while they are in the maternal plasmodium (Slyusarev 2008: 421). Individuals ready for bisexual reproduction emerge from the plasmodia along special outgrowths directed to the surface of the host’s body and enter the surrounding sea water, where fertilization takes place: spermatozoa enter the water through a special genital opening on the body of the male and then enter the body of the female through her genital opening. Fertilized eggs undergo unequal cleavage, resulting in the formation of a morula-like embryo. From the embryo, a small (body size is about 15 microns), covered with setae, larva is formed, which leaves the mother’s body through the genital opening. After swimming freely in sea water, the larva enters the body of a new host, where its outer ciliated layer of cells is shed, and one or more next-generation plasmodia are formed from internal cells (Ivanova-Kazas 1975: 92; Malakhov 1990: 49; Slyusarev 2008). Thus, orthonectids demonstrate asexual reproduction (by budding of plasmodia), ameiotic parthenogenesis, and bisexual reproduction, in which individuals of the new generation develop completely inside the maternal organism (complete viviparity).

The formation of the embryo sac in angiosperm plants (Magnoliopsida) and the processes of embryo and endosperm formation occurring inside this single multinucleated cell are so well known that there is no need to dwell on them in more detail here. However, it is worth noting the remarkable and rather strange circumstance that among plants, embryogenesis according to the “cell within a cell” type appears only in this youngest, evolutionarily advanced group, while the analogous examples listed above among animals are characteristic only of very simply organized groups that have passed to parasitism.

## ﻿Synchronization of copulative processes

Based on the well-known evolutionary advantages of sexual reproduction over asexual reproduction (see, for example, Felsenstein 1974), in order to increase variability and biological diversity, it is theoretically more advantageous for a living organism to reproduce by gametes and their fusion products (zygotes) than by spores. It is well known that the onset of the reproductive period in the life cycle of a particular species of living organisms is somehow coordinated with various environmental factors (see, for example, a review on algae in Brawley and Johnson 1992). However, for reproduction by zygotes, it is necessary not only to synchronize the period of gametogenesis, but also the very moment of gamete release in different individuals of the population, so that “male” and “female” gametes meet in a certain place in space at the same time. The distances that the gametes themselves are able to overcome solely due to cellular movements are very short. This issue has been studied in detail, for example, in diatoms (Davidovich et al. 2012; Davidovich 2019: 151–162). Their “male” gametes, due to flagellar activity, amoeboid movement, or the formation of special cytoplasmic filaments, can move at distances only several times (rarely ten times) greater than the diameter of the gametes themselves. The movement of “male” gametes is chaotic in this case, and the “female” gametes of diatoms are not at all capable of active movement. Thus, for copulation it is necessary that the parental individuals are in close proximity to each other. Often, even as a result of the close but unfortunate location of parental individuals, their gametes cannot merge with each other (Davidovich 2019: 152). However, in general, for microscopic unicellular organisms that form dense populations, the synchronization of gamete release does not seem to be a significant problem (Brawley and Johnson 1992). The gametogenesis of unicellular organisms is just a direct transformation of an “adult” unicellular organism into one or several gametes, takes a relatively short time, comparable to the viability of gametes, and directly depends on the onset of external factors (the same for all individuals of the population), and the resulting gametes usually similar in size to adults. Thus, at the same point in space at the same time, numerous gametes capable of fusion inevitably appear. In addition, some unicellular organisms form a “syzygy” before copulation, within the closed shell of which fusion takes place (Fig. 5), or the union of gametes occurs in the cramped space of the internal cavities of the body of the host organism (in parasitic life cycles).

The situation is quite different in multicellular organisms. First, due to the increase in the size of their bodies, each multicellular organism occupies a place in space that is many times greater than the size of the gametes it produces. Secondly, before the start of gametogenesis, such an organism must reach the complex multicellular stage of the “vegetative” body (see the first reproductive criterion of multicellularity above), which creates a certain (often very significant) individual variation in terms of readiness for the sexual process and maturation of gametes. Thirdly, the appearance of oogamy in multicellular organisms leads to the fact that only male gametes retain their own mobility (and sometimes it is lost in gametes of both sexes). Considering all these features, it is possible to achieve cross-fertilization of gametes at the multicellular level of organization in the following ways: 1) by keeping immobile female gametes in the body of the mother’s organism until they are found by spermatozoa (internal fertilization in the broad sense); 2) forcibly ejecting female gametes into the external environment synchronously with the ejection of spermatozoa by other individuals of the population (external fertilization); 3) providing a passive release of numerous gametes into the external environment at a strictly defined time (also external fertilization).

**Figure 14.**
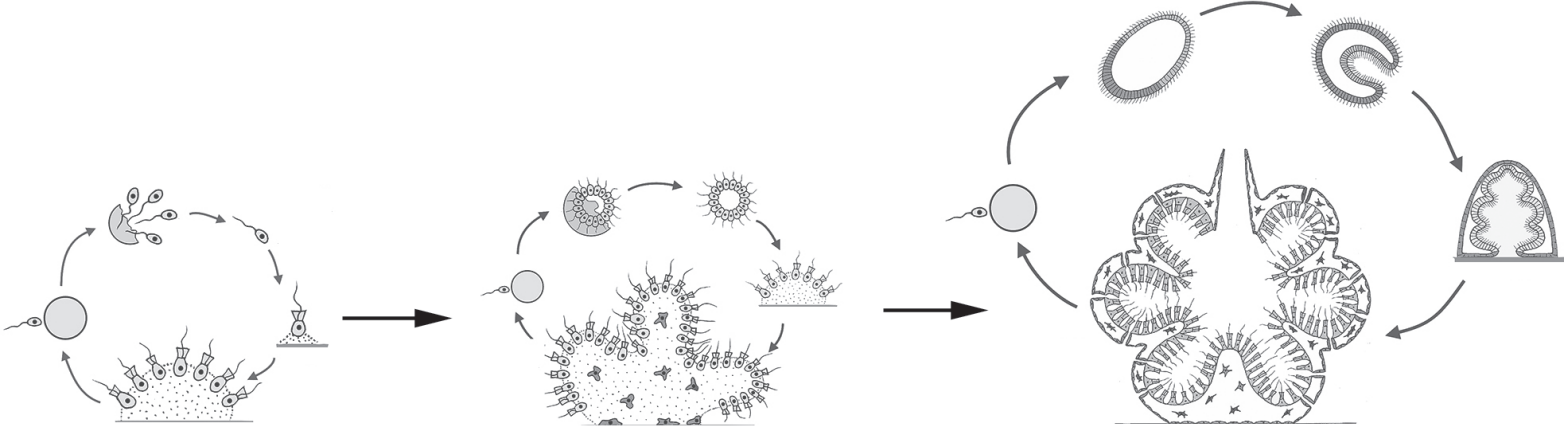
Graphical interpretation of the “sedentary” hypothesis of the origin of multicellular animals under the assumption of the initial external fertilization (according to Malakhov et al. 2019, with changes).

**Figure 15.**
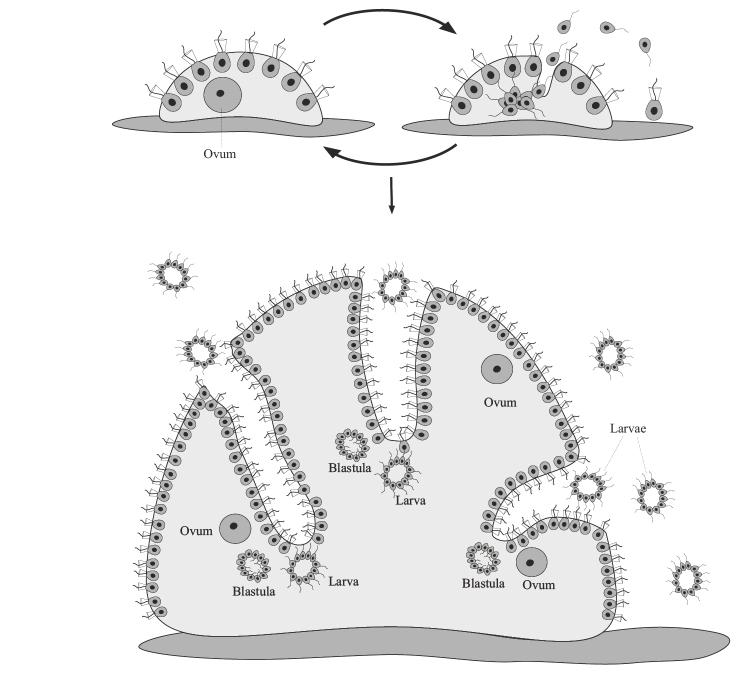
Scheme of the origin of multicellular animals (Metazoa), based on the hypothesis of primary viviparity. Maximal figure size, please!

The first way, undoubtedly, turns out to be technically simpler and is implemented independently in the vast majority of groups of archaic multicellular organisms. Thus, in the vast majority of sponges, in trichoplax, in archaic turbellarians, in extremely simplified orthonectids and dicyemids, in multicellular fungi, in volvox, in most oogamous multicellular algae, as well as in all higher plants, internal fertilization of the egg occurs, and the initial stages of embryogenesis take place inside the body of the mother organism, or the zygote becomes a resting stage and finds itself in the external environment after the death and disintegration of the mother’s body. A clear understanding of this circumstance allows us to answer the age-old question of classical biology about whether for animals and other multicellular organisms the original method of reproduction was external fertilization with the corresponding complete development of the daughter organism in the external aquatic environment. In many old and modern general theoretical works, this was taken for granted so much that it was not even specially argued (see, for example, Franz 1924; Ivanov 1968; Ivanova-Kazas 1995; Mikhailov et al. 2009; Malakhov et al. 2019, etc.). According to such ideas, in hypothetical diagrams of the origin of the first Metazoa from colonial choanoflagellates (Choanoflagellata), it is usually drawn that the eggs somehow get into the water and are fertilized by spermatozoa there (Fig. 14). However, already from the fact that all animals and, in general, all organisms with embryogenic multicellularity are characterized by obligate oogamy, it follows that the female gametes themselves cannot get out of the multicellular body or even from the colony of protists in any way, but the genital ducts, muscles and nervous system, which would regulate the forced release of gametes, are absent yet in archaic organisms; all these apomorphies appear in evolution, starting from the level of organization of coelenterates and above, i.e. in Eumetazoa (for a more detailed analysis, see Gavrilov-Zimin 2022). It remains theoretically possible to passively release oogametes through a simple rupture of the body wall or the cell wall of some conditional “gametangium”, according to the principle of opening an abscess. In this way, for example, various gametangia and sporangia are opened in lower and higher plants. However, this method is not entirely suitable for ensuring fertilization, since each abscess or sporangium is opened at the moment when it is ripe, not in accordance with other abscesses on the body of the same organism, and even more so of another organism. Therefore, contrary to popular belief, all the most archaic multicellular organisms are characterized by internal fertilization of the egg directly inside the body or on the body of the mother’s organism. This means the inevitability of initial viviparity in Metazoa and sporophyte germination on gametophyte in plants (see: Gavrilov-Zimin 2022). That is, the scheme for the origin of animals should look like this (Fig. 15). At first, in some choanoflagellates, the immobile zygote underwent divisions, remaining inside the colony, and the products of this division, mobile zoospores, left the colonies, floated away and gave rise to new organisms. Then the colonies began to grow, the mobility of a single zoospore was no longer enough to leave the huge colony and sail somewhere to a new place. Under these conditions, the appearance of a synzoospore (i.e., a product of zygote cleavage that has not disintegrated into parts), a hypothetical stage of development used by Zakhvatkin (1949; 1956) in his theory of the emergence of multicellular Metazoa, turns out to be logical. In fact, the synzoospore was the first larval stage in the evolution of organisms, which was unable to feed itself yet, but ensured distribution. Such variant of reproduction/development is known, for example, in modern sponges (Porifera).

In some oogamous multicellular algae, due to the simplicity of their structure, it turns out to be rather difficult to draw a clear line between fertilization inside the mother’s body and on its surface. Thus, in Laminariales brown algae, the egg is released from the oogonium before fertilization, but remains attached to its edges. The zygote germinates without detaching from the maternal gametophyte. If, due to random events, the egg or zygote loses its connection with the mother plant, then differentiation processes are disrupted during germination and the resulting defective thallus soon dies (Belyakova et al. 2006b: 133–134).

External fertilization is well known and studied in many groups of marine and freshwater animals, in which this process is ensured by the presence of the nervous system, sensory organs, muscles, and various genital ducts. Receiving a certain signal (visual, tactile, chemical) from each other, sexual partners implement a forced synchronous release of gametes into the external aquatic environment. However, for the most archaic animals and plants, the only possible way is the passive release of gametes, in particular, through the rupture of the shells of the “gametangia”, synchronized by external causes. A comparative analysis of the reproductive strategies of various multicellular organisms shows that it is extremely difficult to achieve synchrony with the passive variant. This path was realized only in a few small groups of marine organisms, strictly synchronized in their reproductive activity with the lunar cycles and/or the corresponding periodicity of tides. In this case, the passive release of gametes is technically provided in two different methods, but both of them are associated with significant limitations and remain evolutionarily dead ends.

The first method is known in some highly developed Demospongiae, which are built according to the progressive “leucon” type and reach large body sizes. The structure of the body allows these sponges to regulate the flow of water passing through the body and carrying out a large number of immobile eggs and motile spermatozoa (Reiswig 1970a, b; 1976; Ereskovsky 2005: 55–59). However, in most cases it remains unknown whether the release of eggs from the body occurs before fertilization or after fertilization (Reiswig 1976: 104). Sponges have no formed gonads; female germ cells are located diffusely or in small groups among the somatic cells of the body, and spermatozoa are collected inside temporary formations – spermatocysts (Ivanova-Kazas 1975; Ereskovsky 2005). This circumstance undoubtedly facilitates the task of excreting gametes with water flows, since there is no need to open the shell of the conditional “gametangium”, which is present in most other multicellular organisms. Pheromones probably act as an additional regulator of the synchronicity of gamete release in sponges.

**Figure 16.**
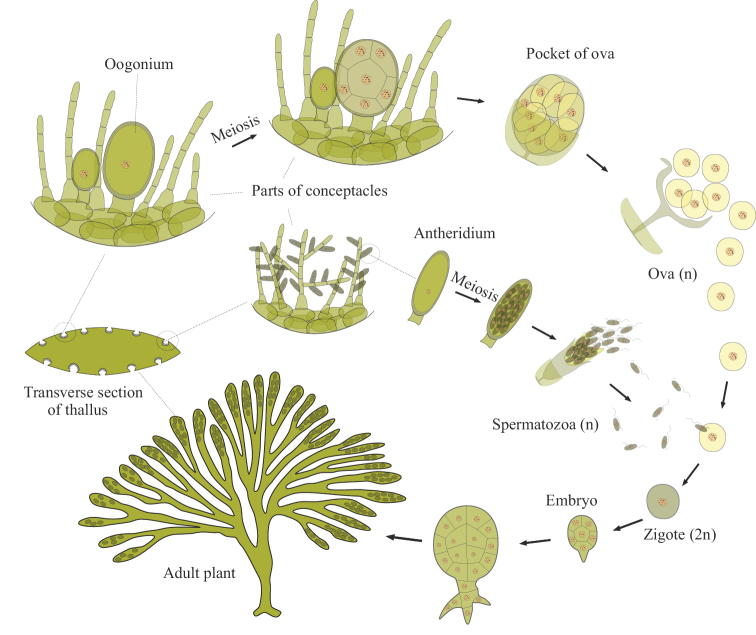
Generalized diagram of the life cycle of *Fucus* spp.

The second way of passive synchronous release of gametes is implemented in a number of genera of brown and green algae. They are unable to regulate water flows, but their reproduction is coordinated in a complex way with the lunar cycle and tidal rhythms (Smith 1947; Brawley 1992; Brawley and Johnson 1992; Feis 2010; Heesch et al. 2021). This synchronization is best studied in various *Fucus* spp. Unlike the vast majority of other plants, meiosis in *Fucus* spp. occurs during the formation of eggs and spermatozoa, there is no gametophyte stage and the haploid phase is represented only by gametes (Fig. 16), similar to how it occurs in the life cycle of animals. The immobile eggs are released into the water simply through a break in the wall of the gametangium and then settle to the bottom, while the motile spermatozoa find them due to pheromones that act at a distance of only a micrometer to a millimeter (Serrão et al. 1996; Feis 2010). That is, fertilization is possible only between algae located next to each other. The synchronism of the release of gametes is achieved due to the fact that the gametangia dry up at low tide, and then massively burst upon repeated wetting and/or changes in salinity during high tide (the so-called osmotic stress). At the same time, it is necessary that the tide be calm, without strong waves that can spread the gametes in different directions. It is clear that this method of bisexual reproduction, based on a combination of many specific external causes, is not suitable for most other living organisms. However, the superficial similarity of the reproductive biology of fucuses with the reproductive behavior of oviparous animals even gave rise at one time to the hypothesis of the origin of the Metazoa directly from fucus-like ancestors (Franz 1924). Extremely accurate synchronization of maturation and excretion of gametes due to tidal rhythms is also known in those algae that retain the alternation of gametophyte/sporophyte in their cycle. In this regard, various species of brown algae of the genus *Dictyota* Lamouroux, 1809 (see Hoyt 1927; Bogaert et al. 2020) and green algae of the genus *Ulva* Linnaeus, 1753 (see Smith 1947) are the most studied.

In laboratory conditions, it is very often possible to achieve synchronous opening of gametangia due to a sharp change in illumination (see review in Brawley and Johnson 1992: 237–238), but such studies were again carried out on algae, whose reproduction under natural conditions is confined to tidal cycles.

There does not seem to be any other effective means of precise synchronization of gamete release, apart from tidal, in multicellular plants. Understanding this, one can offer an explanation for why plants do not have egg-laying, similar to that of animals, and why asexual reproduction with sporophyte/gametophyte alternation absolutely predominates in plants, despite the obvious evolutionary advantage of bisexual reproduction and the diploid state of the multicellular body. The answer lies in the fact that plants are not able to independently release eggs into the external environment synchronously with spermatozoa. Their eggs in the vast majority of cases remain on the mother’s body, wait until spermatozoa (or sperm) reach them in one way or another, and then germinate inside or on the body of the mother’s body. In this case, reproduction and distribution are not provided by gametes or zygotes, but by spores, since no synchronization is required for this at all. Up to the highest stages of plant evolution, they fail to switch to normal independent sexual reproduction, and most flowering plants in their sexual process are also completely dependent on animals, especially pollinating insects. There are examples of plant gamete transfer in some marine plants, for example, in some red algae, for which crustaceans act as pollinators (Ollerton and Ren 2022).

In animals, on the contrary, bisexual reproduction absolutely predominates, and synchronization of the release of gametes is achieved at fairly early stages of their evolution, starting with the most complexly organized sponges and coelenterates. The latter develop a simple nervous system, gonads, and musculature, in particular, a muscular intestine/stomach, through which, in the simplest case, sexual products are excreted. Some ctenophores (Ctenophora) even have specialized reproductive ducts (Beklemishev 1964: 334). As a result, both among cnidarians (Cnidaria) and among ctenophores (Ctenophora), external fertilization with the development of eggs in the external environment prevails in the vast majority of species, and only a few species retain viviparity or ovoviviparity. All further evolution of the reproductive sphere of animals is the constant improvement of the reproductive ducts, gonads, external ovipositors and copulatory organs, and the very methods of laying eggs protected by shells into the external environment. Separate aberrations of the reproductive system, leading in some groups, small in diversity, to secondary vivparity, I have considered in detail in a special article (Gavrilov-Zimin 2022).

## ﻿Policytic reproduction

The simplest version of polycytic reproduction, which consists in restoring the whole body from separate fragments, is observed in almost all archaic multicellular organisms and probably represents the original (plesiomorphic) method of polycytic reproduction for most phylogenetic lines. Despite its extreme archaism, the ability to restore the whole body from fragments is retained during the entire further evolution in most groups of plants, including the most highly developed angiosperms (Magnoliopsida), as well as in most fungi. On the contrary, among animals, this method remains possible only in organisms that are at a relatively low level of morpho-anatomical organization: sponges (Porifera), coelenterates (Coelenterata), various taxa of flatworms (Plathelminthes), some nemerteans (Nemertini), and annelids (Annelida). A somewhat more complicated version of fragmentation can be considered the division of the body in two by lacing or splitting. Such methods are known, for example, in trichoplax, some coelenterates and flatworms. At the same time, division without previous morphogenetic preparation (architomy) and division after preliminary doubling of body parts (paratomy) are distinguished – see, for example, Zakhvatkin (1949: 171).

**Figure 17.**
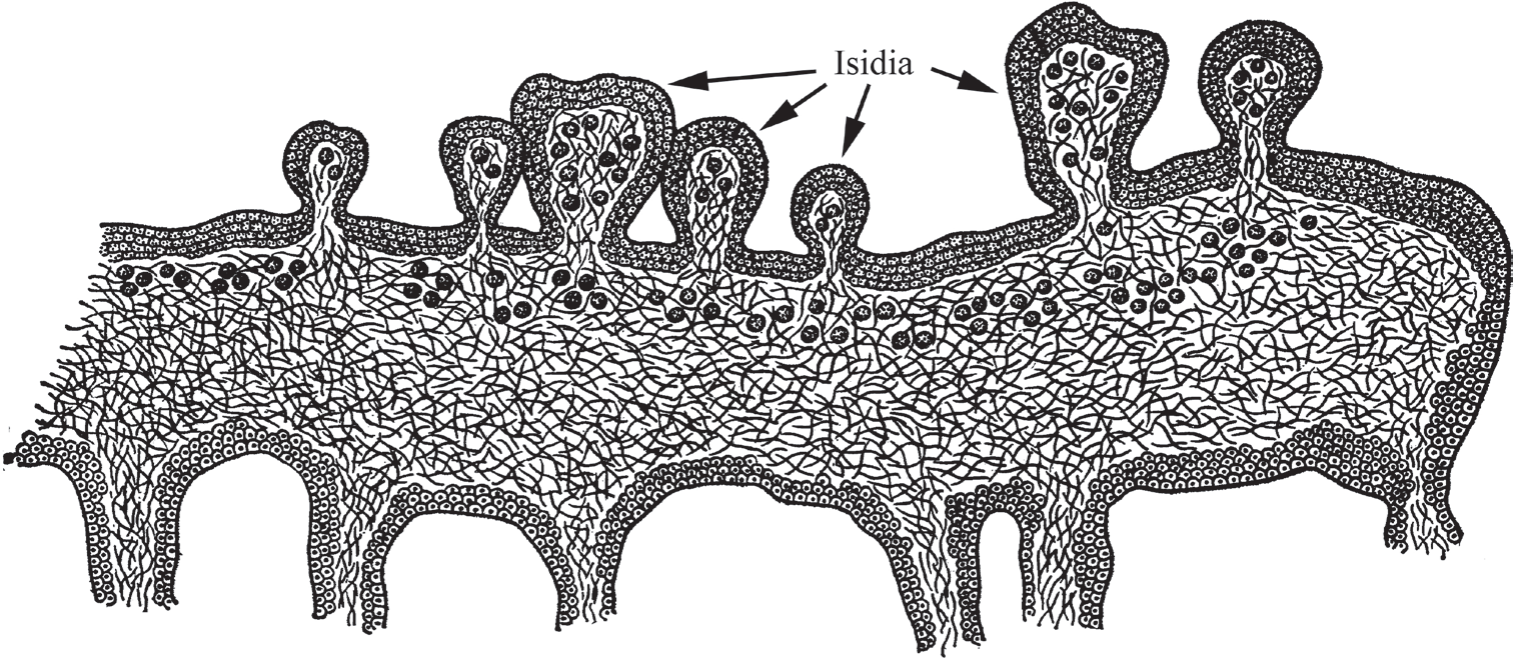
Polycytic budding in lichens: reproduction by isidia (after Golubkova 1977, with changes).

An apomorphic feature inherent in some protonemal and embryogenic multicellular organisms, as well as representatives of “complex” organisms – lichens (Lecanoromycetes) — can be considered the appearance in them of a special polycytic budding (= blastogenesis), as a result of which specialized outgrowths are regularly formed from groups of somatic cells, over time, separating and growing into independent individuals. In many groups of organisms, such polycytic budding occupies a strictly defined place in the life cycle or even represents the main way of reproduction and distribution in space. So, in many highly organized representatives of lichens, polycytic budding is the only way of reproduction (not counting accidental fragmentation of the body). This process is carried out through the formation of the so-called soredia and isidia (Fig. 17) – microscopic multicellular outgrowths of the thallus that combine symbiotic fungal hyphae and algae cells (Golubkova 1977: 419; Belyakova et al. 2006a: 224–226).

Polycytic budding is highly developed in Charophyceae s.s. and is provided by special nodules on rhizoids or by special “stellate cell clusters” (Belyakova et al. 2006b: 270).

A significant diversity of polycytic “brood bodies” is observed in gametophytes of various liverworts (Marchantiophyta) and mosses (Bryophyta) (Abramov and Abramova 1978: 65–66; 81–82; Potemkin and Sofronova 2009: 30). Brood nodules and buds are known in sporophytes of some Lycopodiophyta (Filin 1978: 106, 114) and Psilotopsida (Timonin and Filin 2009: 298). The sporophytes of many horsetails (Equisetopsida) are characterized by the formation of numerous underground nodules (Filin 1978: 140–141). Brood nodules and buds are known in sporophytes of some species of ferns (Pteridiophytina) (Timonin and Filin 2009: 255). However, polycytic budding is most common among flowering plants (Reproductive Systems 2000: 315).

**Figure 18.**
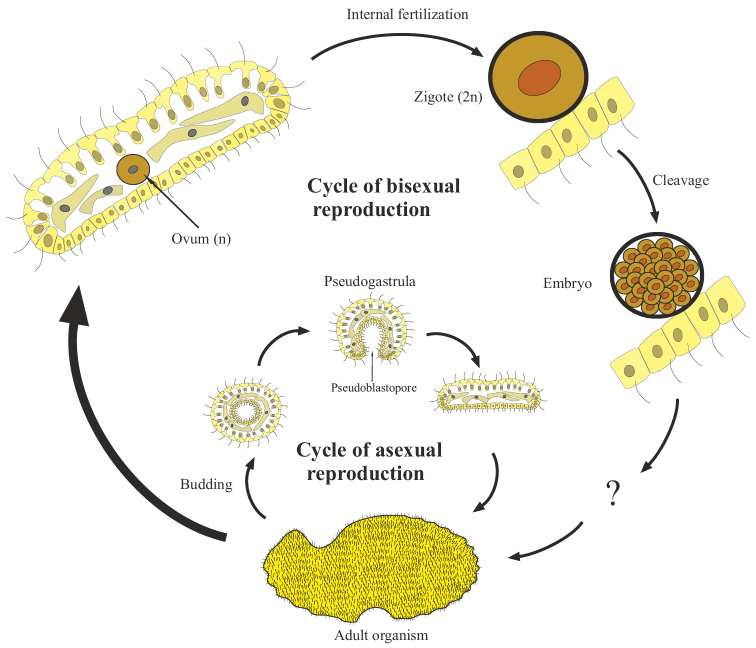
Scheme of the life cycle of *Trichoplaxadhaerens* Schulze, 1883; asexual reproduction is provided by polycytic budding.

In animals, polycytic budding is widespread among sponges (Porifera), trichoplax (Fig. 18), cnidarias (Cnidaria), flatworms (Plathelmintes), camptozoa (Kamptozoa), annelids (Annelida), many tentaculata (Tentaculata) and hemichordates (Hemichordata), a number of lower chordates – tunicates (Tunicata), some species of echinoderms (Echinodermata) and, in rare examples, are known from representatives of some other groups (Ivanova-Kazas 1977, 1995).

Polyembryony can be considered a special type of polycytic budding, apomorphic for some embryogenic multicellular organisms. This term, like many others used in reproductive biology, has a rather vague meaning. In most cases (and in this article), polyembryony means the regular division of a developing zygotic embryo into several secondary embryos (see, for example, Ivanova-Kazas 1995: 205). At the same time, in many embryogenic multicellular organisms, the division of the developing embryo into separate blastomeres is possible by chance or experimentally induced. So, for example, in some cases facultative “primary polyembryony” is noted, which manifests itself in experiments and is the cultivation of independent organisms from individual blastomeres, for example, in some hydromedusae (Zakhvatkin 1949: 217; Ivanova-Kazas 1977: 200–201). In my opinion, in this case we are not talking about polyembryony in the sense mentioned above, but only about the forced “reconstruction” of that stage of the life cycle that took place in the unicellular ancestors of the organisms under consideration, namely, the division of the zygote into separate zoospores.

Polyembryony is extremely widely understood in the literature on flowering plants (Reproductive Systems 2000: 401), where it is proposed to use this term not only for fairly rare cases of regular division of the initial zygotic embryo (as, for example, in peonies (*Paeonia* spp.), but also various cases the emergence of germ-like structures from vegetative parts of the body. I am not ready to agree with such an expansive approach, since it generates terminological confusion.

Rare cases of polyembryony among animals are known in some genera of cyclostomes (Cyclostomatida), monogenetic flukes (Monogenea), endoparasitic hymenopterans (Hymenoptera) and Strepsiptera, as well as in mammals – armadillos of the genus *Dasypus* Linnaeus, 1758 (Ivanova-Kazas 1995: 205, 208, 257, 271, 275, 475, 480).

An extremely peculiar analogue of polyembryony can be seen in the development of the so-called “carposporophyte generation” of red floridian algae (Rhodophyta: Florideophyceae) (Fig. 8). In most floridias, the “zygote” (the fertilized “carpogon”), one way or another, merges with the “auxiliary” (nourishing) cells of the maternal thallus (gametophyte). After fusion, carpogon forms numerous multicellular processes (“gonimoblast threads”). Then all or only part of the gonimoblast cells become “carpospores”. After separation from the mother plant, carpospores give rise to the next diploid generation (tetrasporophytes) (Vinogradova 1977a: 192–250; Searles 1980). If we accept the idea of an analogy of polyembryony with respect to floridias, then the need for such a complicated theoretical construction as an additional generation of sporophytes (“carposporophyte generation”) disappears, and the life cycle of red algae then turns out to be quite comparable with the usual gametophyto-sporophyte cycle of other plants.

A number of groups of multicellular organisms completely lose the ability for polycytic reproduction (with the exception of the rarest cases of polyembryony mentioned above). Such, for example, are various taxa within the polyphyletic group of Nemathelmintes, echiurids (Echiurida), brachiopods (Brachiopoda), arthropods (Arthropoda), mollusks (Mollusca), vertebrates (Vertebrata). Obviously, such a loss is associated with a high degree of specialization of the tissues and organs of these organisms and the corresponding loss of totipotency in most of the somatic cells that make up their body. At the same time, the almost total absence of polycytic reproduction in gymnosperms and even in such simply organized multicellular plants as *Volvox* spp. is not entirely clear.

## ﻿Conclusion

The multiple origin of multicellularity in different groups of organisms allows at the present time to give only a very approximate minimum estimate of the total number of such evolutionary events. Apparently, there were at least 50 cases of independent origin of multicellularity among eukaryotes and at least several dozens among prokaryotes. Examples of protonemal multicellularity among bacteria and algae are of particular difficulty for calculation, since the modern systems of these organisms abound in genera that simultaneously include species with simple unicellular, colonial-unicellular and obligate-multicellular bodies (see, for example, AlgaeBase: https://www.algaebase.org/). It is equally difficult to count the numerous cases of transition from siphon-unicellular to siphonoseptal multicellularity among fungi and algae, developing through the initial stage of a multinuclear “siphon”. A much clearer picture emerges with regard to embryogenic multicellular organisms. Thus, there is no doubt about the single independent appearance of animals and separately *Volvox* spp. on the basis of the corresponding ancestral spherical colonies with an internal cavity (Zakhvatkin 1949; Malakhov et al. 2019, etc.). The single occurrence of higher plants (Embryophyta) and charophyceae algae (Charophyceae s.s.) based on the preceding protonemal multicellularity of their ancestral forms is also generally accepted in the botanical literature (see, e.g., Umen 2014). It is believed that sporophytes of higher plants in all cases develop embryogenically, while gametophytes in many cases retain protonemal development. Embryogenic multicellularity among brown and red algae, apparently, arose repeatedly, but on the basis of the already achieved protonemal multicellularity of more archaic representatives of these groups (see above).

It is noteworthy that all complex multicellular organisms that have tissues and organs develop according to the type of embryogenic multicellularity based on obligate accumulative oogamy or accumulative aplanosporia. This is probably due to the well-known fact that a large volume of cytoplasm in the egg and its complex structure are very important for the initial differentiation, which then ensures the predetermination of cleavage and the formation of specific tissues and organs from certain blastomeres. For animals, in addition to the initial predetermination of cleavage, the formation of internal body cavities, in particular, the primary cavity (the blastocoel), is also important, and this, probably, cannot be achieved on the basis of protonemal or siphonoseptal development.

Summing up all of the above, I can highlight the following final suggestions:

1. The proposed first reproductive criterion of multicellularity postulates that a unitary multicellular organism, in contrast to a colonial-unicellular organism, obligately develops as a multicellular organism and reproduces itself only after it reaches the multicellular “vegetative” stage of ontogenesis.
2. The second reproductive criterion of multicellularity determines exactly how a multicellular body reproduces itself in course of the monocytic method and allows us to divide all known ways of implementing obligate multicellularity into three fundamentally different options: protonemal, siphonoseptal and embryogenic.
3. The most complex, embryogenic multicellularity arises exclusively on the basis of obligate accumulative oogamy or accumulative aplanosporia, in which the gamete / spore exceeds in size (sometimes hundreds and thousands of times) the original mother cells. As a result of subsequent palintomic or syntomic divisions, an embryo or embryoid is formed from an oogamete/spore — stages of ontogenesis that are absent in other multicellular and unicellular organisms.
4. The emergence of multicellularity, especially on the basis of oogamy, creates significant technical problems for the synchronization of copulatory processes. The simplest way out of this situation is to keep immobile female gametes in/on the body of the maternal organism until they are found by spermatozoa. This method is implemented in the vast majority of multicellular plants and fungi, as well as in the most archaic animals. In this regard, viviparity is considered as the original, plesiomorphic way of offspring in Metazoa.

## Footnotes

In green algae of the genus Volvox Linnaeus, 1758, s.l. (Chlorophyceae: Chlamidomonadales) embryogenic multicellularity is realized, while in the genus Sphaeroplea Agardh, 1824 (Chlorophyceae: Sphaeropleales) siphonoseptal multicellularity occurs (see below).
